# Towards Stewardship of Wild Species and Their Domesticated Counterparts: A Case Study in Northern Wild Rice (*Zizania palustris* L.)

**DOI:** 10.1002/ece3.71033

**Published:** 2025-03-13

**Authors:** Lillian McGilp, Matthew W. Haas, Mingqin Shao, Reneth Millas, Claudia Castell‐Miller, Anthony J. Kern, Laura M. Shannon, Jennifer A. Kimball

**Affiliations:** ^1^ Department of Agronomy and Plant Genetics University of Minnesota St. Paul Minnesota USA; ^2^ Department of Plant Pathology University of Minnesota St. Paul Minnesota USA; ^3^ Department of Math, Science and Technology University of Minnesota Crookston Minnesota USA; ^4^ Department of Horticultural Science University of Minnesota St. Paul Minnesota USA

**Keywords:** domestication, genetic diversity, genotyping‐by‐sequencing, selection, spatio‐temporal diversity, *Zizania palustris*

## Abstract

Northern Wild Rice (NWR; *Zizania palustris* L.) is an aquatic, annual grass with significant ecological, cultural, and economic importance to the Great Lakes region of North America. In this study, we assembled and genotyped a diverse collection of 839 NWR individuals using genotyping‐by‐sequencing (GBS) and obtained 5955 single‐nucleotide polymorphisms (SNPs). This collection consisted of samples from 12 wild NWR populations collected across Minnesota and Western Wisconsin, some of which were collected over two time points; a representative collection of cultivated NWR varieties and breeding populations; and a *Zizania aquatica* outgroup. Using these data, we characterized the genetic diversity, relatedness, and population structure of this broad collection of NWR genotypes. We found that wild populations of NWR clustered primarily by their geographical location, with some clustering patterns likely influenced by historical ecosystem management. Cultivated populations were genetically distinct from wild populations, suggesting limited gene flow between the semi‐domesticated crop and its wild counterparts. The first genome‐wide scans of putative selection events in cultivated NWR suggest that the crop is undergoing heavy selection pressure for traits conducive to irrigated paddy conditions. Overall, this study presents a large set of SNP markers for use in NWR genetic studies and provides new insights into the gene flow, history, and complexity of wild and cultivated populations of NWR.

## Graphical abstract

Analysis of Northern Wild Rice ( *Zizania palustris* ) wild and domesticated populations reveal little to no gene flow between the two as well as insights into the historical management of wild populations.

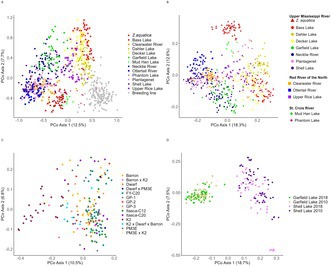

## 1 Introduction

Northern Wild Rice (NWR; *Zizania palustris* L.) is an annual, diploid (2*n* = 2*x* = 30), aquatic grass native to the Eastern Temperate and Northern Forest ecoregions of North America (Elliott 1980; Grombacher et al. 1997). Predominantly found in shallow, slow‐moving waters surrounding the Great Lakes region of North America, NWR's regional significance in these areas is vast and complex (Dean Biesboer 2019; Desmarais 2019; Drewes and Silbernagel 2005; Lu et al. 2005; Pollman et al. 2017; de Wet and Oelke 1978). To begin, NWR is a natural resource that provides food and substantial habitat for a wide range of wildlife (Fannucchi 1983; Moyle 1944) as well as important ecosystem services such as anchoring riparian soils and inhibiting algal blooms (Rogosin 1954). For centuries, this nutritious grain has been hand‐harvested from regional lakes and rivers by Dakota and Anishinaabe Peoples (Grombacher et al. 1997; Matson et al. 2021), and “psin” (Dakota) or “manoomin” (Ojibwe, Anishinaabe) remains an integral component of their cultures and lives today. The species has also become a high‐value commodity crop that includes hand‐harvested grain from regional waters, and cultivated grain from irrigated paddies, grown primarily in Minnesota (MN) and California (CA) (Oelke et al. 1982). As a part of the *Oryzae* tribe in the *Poaceae* family, *Zizania* species are also considered crop wild relatives of *Oryza sativa* L. (white rice). Given the many roles described above, we contend that the conservation of NWR serves as an important intersection between our ecosystems, our cultures and food, and our economies.

Worldwide, plant species are experiencing declines and extinction events as a result of human activities altering natural ecosystems. For at least the last century, NWR has been experiencing such declines in its native habitats (Hansen 2008; Norrgard 2006), and the species appears to be slowly migrating northward (Terrell et al. 1997). Recent reports have stated that NWR is at high risk of loss, and the International Union for Conservation of Nature has added NWR to its red list of threatened species (Maiz‐Tome 2016). Hydrological changes due to damming and channelization, recreational water activity, shoreline development, and water pollution from industrial activities have all been associated with the decline of NWR in its natural habitats (Hansen 2008; Myrbo et al. 2017). The species is particularly sensitive to high levels of sulfates in its water supply and acts as an important indicator species of water quality (Myrbo et al. 2017; Fort et al. 2014). In addition to these conservation challenges, NWR seed is considered intermediate or desiccation intolerant, which reduces seed longevity in storage to 1–2 years (Probert and Longley 1989) and complicates the species' preservation in *ex situ* seed banks. Alternate storage options including variations in temperature, partial drying, dry or moist storage, and cryopreservation have been attempted with limited success (Aldridge and Probert. 1992; Kovach and Bradford 1992; McGilp et al. 2020). As such, the extent of the genetic diversity of NWR is primarily preserved in situ within its natural range (Porter 2019), lacking the safeguards that *ex situ* genetic reservoirs can provide.

Genetic diversity represents the extent of heritable variation within and among populations of a species, and its preservation is vital for the maintenance of long‐term viability in the face of continual environmental change (Ellegren and Galtier 2016; Frankel 1970). A 2008 MN Department of Natural Resources (MN DNR) report on the health of NWR natural stands concluded that the species' greatest threat was an overall state‐wide decline in genetic diversity (Hansen 2008). Biologists and conservationists have widely recognized the value of characterizing genome‐wide diversity within a species for use in conservation efforts (Santamaría and Méndez 2012; Sgrò et al. 2011). However, few such studies have been conducted for NWR, and molecular studies have heavily relied on older marker systems (Lu et al. 2005; Diller et al. 2018; Kahler et al. 2014), which are laborious to produce and often limited in number (Collard et al. 2005). The advent of low‐cost high‐throughput sequencing, such as genotyping‐by‐sequencing (GBS), which provides genome‐wide coverage of co‐dominant, single‐nucleotide polymorphism (SNP) markers, has improved the genetic diversity characterization of extensive and complex germplasm collections (Elshire et al. 2011). In 2019, a study with a limited sample size demonstrated the potential for GBS to be applied to NWR (Shao et al. 2019).

The cultivation of NWR in irrigated man‐made paddies, similar to white rice production, began in the 1950s to create an industry capable of supplying a consistent source of grain to agricultural markets. As such, the production of cultivated NWR (cNWR) is a fairly new endeavor, and only ~60 cycles of targeted selection separate cNWR from its wild counterparts. Breeders of cNWR have focused primarily on adapting the species to agronomic production in paddies and on improving seed retention in crop (Grombacher et al. 1997). However, concerns regarding the potential impact of gene flow between cNWR and natural stands of NWR have been raised given the species' out‐crossing nature (Matson et al. 2021; Gross 2021; Raster and Hill 2017; Streiffer 2005). The Great Lakes region is the center of both the origin and diversity of *Z. palustris* ; therefore, it is important to understand the potential impact of domesticating and cultivating cNWR in those areas. As plant breeders, we have a responsibility to be good stewards of our natural and domesticated plants. Thus, an understanding of how cNWR fits into local landscapes and, particularly, to what extent gene flow is occurring among natural stands and cNWR is essential to overall germplasm preservation.

In this study, we generated a genome‐wide SNP dataset via GBS for a NWR diversity collection to study the population structure and gene flow within and among wild and cultivated populations. We aimed to improve our understanding of the genetic variability within the species and provide new information regarding the selective pressures applied to cNWR germplasm.

## 2 Materials and Methods

### 2.1 Plant Materials

The diversity collections consisted of wild populations gathered across northern MN and Wisconsin (WI) (referred to here as the Natural Stand collection); cultivars and germplasm from the UMN cNWR breeding program (referred to here as the Cultivated collection); and a population of *Zizania aquatica* L. from the Platte River in MN (referred to here as the outgroup). A Temporal panel was also generated to look at potential changes in diversity over time within two natural stand lake populations. In all, a total of 889 individuals were evaluated in this study, which consisted of 530 Natural Stand and 209 Cultivated samples collected in 2018, in addition to 100 samples collected in 2010 for the Temporal panel.

**FIGURE 1:**
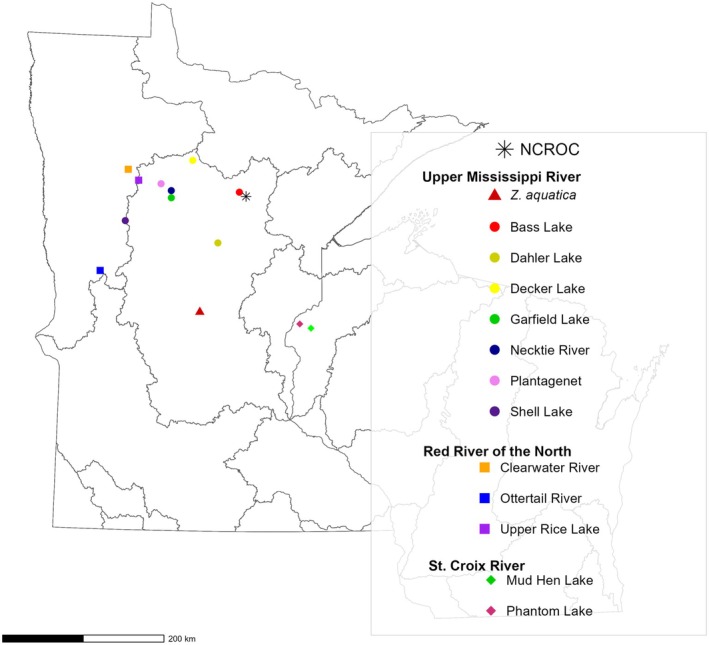
A Hydrological Unit Code‐8 (HUC‐8) watershed map of Minnesota and western Wisconsin indicating areas of sample collection of Northern Wild Rice (NWR; *Zizania palustris* L.) and *Zizania aquatica* L. GPS coordinates can be found in Table S1.

Leaf tissue samples were collected from 10 wild populations across northern Minnesota (50 samples per lake/river) and 2 wild populations from western Wisconsin (10 from Mud Hen Lake; 20 from Phantom Lake) (Figure 1; Table S1). An additional 50 *Z. aquatica* samples, also collected in central Minnesota, were used as an outgroup in this study. Two 8 cm leaves per individual per population were collected and stored in plastic bags on ice until further processing. Natural Stand population samples were collected on parallel transects, with ≥ 10 m intervals between sampled individuals to avoid sampling siblings. Three major hydrologic unit code (HUC) subbasins were represented in this collection, including seven populations from the Upper Mississippi River (UMR) watershed, three populations from the Red River of the North (RRN) watershed, and two populations from the St. Croix River (SCR) watershed (Figure 1; Table S1). A distance (km) matrix for all Natural Stand populations is available in Table S2.

The Cultivated collection consisted of leaf samples from 209 open‐pollinated individuals, representing 4 cultivars and 10 breeding populations from the UMN cNWR breeding program. Samples were collected at the UMN North Central Research and Outreach Center (NCROC) in Grand Rapids, MN, in 2018 (Table S1; Figure 1; Figure S1).

Our Temporal collection consisted of 200 natural stand samples collected from Garfield and Shell Lakes in 2010 and in 2018, with 50 samples collected in each lake at each time point. GPS coordinates were used to ensure that the same population at the same location was collected at both time points (Table S1). We would like to note that samples from Garfield and Shell Lakes collected in 2018 were also a part of the Natural Stand collection (50 samples per lake).

### 2.2 DNA Extraction and Sequencing

During collection, approximately 8 cm of leaf tissue was harvested on ice and then lyophilized using a TissueLyser II (Qiagen, Valencia, CA, USA). Genomic DNA was extracted using a Qiagen DNeasy Plant Mini Kit (Qiagen, Valencia, CA, USA) according to the manufacturer's instructions. DNA concentration was measured with a NanoDrop spectrophotometer (Thermo Scientific, Wilmington, DE, USA); samples were submitted to the UMN Genomics Center for library preparation and sequencing. The digestion step was performed using two restriction enzymes, *Btg1* (5′‐C/CRYGG‐3′) and *TaqI* (5′‐T/CGA‐3′) (Shao et al. 2019). Afterward, unique barcodes were ligated to DNA fragments for sample identification and pooling. Single‐end 150‐bp sequencing to a depth of 2.5 million reads per sample was performed on an Illumina NovaSeq machine (Illumina, San Diego, CA, USA). Raw data were deposited in the National Center for Biotechnology Information Short Read Archive (NCBI SRA) under accession number PRJNA774842. BioSample accession numbers for individual samples are provided in Table S3.

### 2.3 Read Mapping

Quality control was initially performed on the FASTQ files using fastQC version 0.11.7 (Andrews 2010) to check for read quality and adapter contamination. After adapter trimming with Cutadapt version 1.18 (Martin 2011), reads were mapped to the reference genome v1.0 of cNWR cultivar “Itasca‐C12” (Haas et al. 2021) of BWA‐MEM version 0.7.13 (Li 2013). SNPs were called using the “mpileup” function from BCFtools version 2.3 (Li 2011) and sorted with the sort function from samtools version 1.9 (Li et al. 2009), resulting in 2183 variant call format (VCF) files, one for each scaffold of the NWR genome (Haas et al. 2021). The 17 largest VCF files representing the 15 NWR chromosomes and 2 additional large scaffolds of the NWR reference genome were merged into a single VCF file with the “concat” function from BCFtools. Filtering of the merged VCF file was carried out using VCFtools (Danecek et al. 2011). All analyses were done with default parameters. A maximum missing rate of 20% across all samples and a minimum depth of 4 reads per variant site were used to obtain the final SNP set.

### 2.4 Marker Statistics

Summary statistics were calculated for each SNP, including their distribution across chromosomes and scaffolds, their polymorphism information content (PIC) value, and their transition/transversion (TsTv) ratio. Polymorphism information content (PIC) was calculated using the *snpReady* R package (Granato et al. 2018). The number and type of transitions and transversions were calculated using VCFtools.

### 2.5 Genetic Diversity Assessments

To assess the structure and distribution of genetic variation within our collections, we conducted a principal coordinate analysis (PCoA). To generate the full sample PCoA, the VCF file resulting from our SNP calling pipeline was imported into the R statistical environment (R Core Team 2021) using the *vcfR* package (Knaus and Grünwald 2017). For the individual Natural Stand, Cultivated, and Temporal PCoAs, the original VCF file was first subsetted to include only the relevant samples using PLINK version 1.90b6.10 (Purcell et al. 2007). For all PCoAs, the R package *vegan* (Oksanen et al. 2022) was used to calculate a dissimilarity matrix based on the Jaccard distance using the “vegdist” function, as well as eigenvectors and eigenvalues using the “cmdscale” function. PCoA plots were then generated using the *ggplot2* package (Wickham 2016).

NeighborNet diagrams for the Natural Stand and Cultivated collections were generated in SplitsTree (Huson and Bryant 2006) using pairwise distances calculated in *poppr* (Kamvar et al. 2014, 2015). Figures were made with individual as well as averaged population level distances. Neighbor‐joining trees for the Temporal collection were created using the R packages *adegenet* (Jombart and Ahmed 2011; Jombart 2008), *ape* (Paradis and Schliep 2019), *poppr*, and *vcfR*. The temporal cluster analysis was performed similarly to the methods described in Jacquemyn et al. (2006). We estimated Prevosti's genetic distance using the unweighted pair group method with arithmetic mean (UPGMA) algorithm option for the “aboot” function (bootstrapped dendrograms) in *poppr* with 1000 bootstrap replicates, and a selected cutoff value of 50.

Population structure and admixture in our Natural Stand and Cultivated collections was assessed using Bayesian clustering implemented in STRUCTURE version 2.3.4 (Pritchard et al. 2000). Genotypic data for these individuals and the final bi‐allelic SNP set were loaded into STRUCTURE and analyzed with the admixture model. The Markov Chain Monte Carlo was run from *K* = 2 to *K* = 14 with a burn‐in length of 1000 followed by 10,000 iterations. Lower *K* values were used to look for larger structural diversity patterns, while the higher *K* values were chosen to test if we were able to separate each population into their own STRUCTURE‐assigned cluster (*K* = 14 or 12 Natural Stand populations, *Z. aquatica* , and one Cultivated group). Each *K* value was run 3 times, then compiled into a merged file using the “clumppExport” function from the *pophelper* R package and subsequently plotted with the *pophelper* package (Francis 2017). The ideal number of clusters was determined using the DeltaK statistic from the Structure Harvester web tool version 0.6.94 (Earl and vonHoldt 2012), which uses the Evanno et al. (2005) method for determining the number of clusters (Evanno et al. 2005).

Analysis of Molecular Variance (AMOVA) was performed using the “poppr.amova” function from the *poppr* R package based on the work of Excoffier et al. (1992). Groups were defined based on their collection membership (e.g., their lake/river of origin or cultivar/breeding line identity), species ( *Z. palustris* or *Z. aquatica* ), and, more broadly, their germplasm type (e.g., Natural Stand or Cultivated). To detect significant groupings, we performed AMOVAs using default parameters in the *ade4* package, including the use of the “farthest neighbor” algorithm due to the genetic structure among NWR populations and limited gene flow identified in previous studies (Dray and Dufour 2007). The significance was determined using the “randtest” function with 999 repetitions.

Correlation between geographic and genetic distances in our Natural Stand collection was assessed with a Mantel test using the “mantel. test” function from the *ape* R package, based on genetic distances calculated with the “dist.genpop” function from the *adegenet* R package and geographic distances calculated using the “distm” function from the *geosphere* R package (Hijmans 2022). The regression equation was found by fitting the genetic and geographic distances to a linear model. The calculated geographic distances were also plotted against *F*st/(1‐*F*st) to make an isolation by distance graph for comparison with the mantel dot plot.

### 2.6 Gene Flow

The migration rates between populations were calculated using the BayesAss3‐SNPs (BA3) software version 3.0.5.7 (Mussmann et al. 2019). BA3 was run with 10 million iterations, a burn‐in of 1 million, sampling of 200, Δ*F* = 0.1, Δ*A* = 0.25, and Δ*M* = 0.1. Pairwise estimations of genetic differentiation (*F*_ST_) (Francis 2017) between different subgroups based on geographic origin and germplasm type (cultivated population, natural stand, species) were also calculated using the “stamppFst” function from the *StAMPP* R package (Pembleton et al. 2013). Dsuite version 0.4 (Malinsky et al. 2021) was used to calculate Patterson's *D*‐statistics, also known as the ABBA‐BABA statistic, in order to test for introgressions between groups. Since this analysis requires four groups, we defined the groups based on their membership in STRUCTURE‐assigned groups when *K* = 4. This approach split the Natural Stands into two groups, while the Cultivated collection formed a third group. The fourth group (the outgroup) was *Z. aquatica* .

### 2.7 Genome‐Wide Scans for Signatures of Selection

We used a series of tests to identify putative selection events in cultivated NWR. We calculated nucleotide diversity (π) (Nei 1987), Tajima's *D* (Tajima 1989), and *F*_ST_ (Weir and Cockerham 1984) for each SNP using VCFtools. Natural Stand and Cultivated collections were analyzed separately. The results were plotted in the R statistical environment by averaging π values of ~10 Mb/bin. We also used the Cross‐Population Composite Likelihood Ratio (XP‐CLR) test (Chen et al. 2010) to test for large deviations between Natural Stand and Cultivated collections.

## 3 Results

### 3.1 Genotyping‐by‐Sequencing

Using GBS technology, 839 *Z. palustris* samples representing the Natural Stand, Cultivated, and Temporal collections, along with 50 samples of *Z. aquatica* , which served as an outgroup, were sequenced to a depth of 2.5 million reads per sample. A total of 1,833,504,458 reads were generated, with an average of 2,185,345 reads per sample. From these data, a total of 5955 SNP markers were identified that met our filtering criteria, 3005 (50.1%), of which reside in genes. Basic marker statistics are shown in Table 1. The SNP density ranged from 1.15 to 6.37 SNPs/Mb per chromosome, with a genome‐wide average of 4.34 SNPs/Mb using 1 Mb bins. In genic regions, the SNP density ranged from 10 to 20 SNPs/Mb per chromosome. The genome‐wide TsTv ratio was 3.15, with a minimum of 1.50 (ZPchr0458) and a maximum of 4.18 (ZPchr0016) (Table S4). PIC ranged from 0.030 to 0.313, with an average of 0.217 (Table S5).

**TABLE 1:** Summary marker statistics for 5955 Northern Wild Rice (NWR; *Zizania palustris* L.) bi‐allelic single‐nucleotide polymorphism (SNP) markers generated via genotyping‐by‐sequencing (GBS).

Chr/Scaffold	Size (Mbp)	SNPs (#)	% of all SNPs	SNPs in genic regions (#)	% of SNPs in genic regions/Chr
ZPchr0001	95.4	483	8.11	74	15.32
ZPchr0002	103.4	435	7.30	56	12.87
ZPchr0003	58.8	302	5.07	37	12.25
ZPchr0004	98.7	491	8.25	24	4.89
ZPchr0005	66.6	350	5.88	41	11.71
ZPchr0006	118.0	598	10.04	126	21.07
ZPchr0007	42.6	181	3.04	66	36.46
ZPchr0008	75.7	367	6.16	38	10.35
ZPchr0009	95.1	481	8.08	25	5.20
ZPchr0010	111.4	568	9.54	75	13.20
ZPchr0011	63.2	285	4.76	41	14.39
ZPchr0012	105.9	410	6.88	86	20.98
ZPchr0013	111.3	583	9.79	66	11.32
ZPchr0014	24.0	91	1.53	28	30.77
ZPchr0015	39.1	237	3.98	12	5.06
ZPchr0016^a^	13.8	88	1.48	6	6.82
ZPchr0458^a^	4.3	5	0.08	3	60.00

^a^ Additional unaligned scaffolds from the NWR reference assembly v1.0 (Haas et al. 2021).

### 3.2 Genetic Diversity of NWR Collections

**FIGURE 2:**
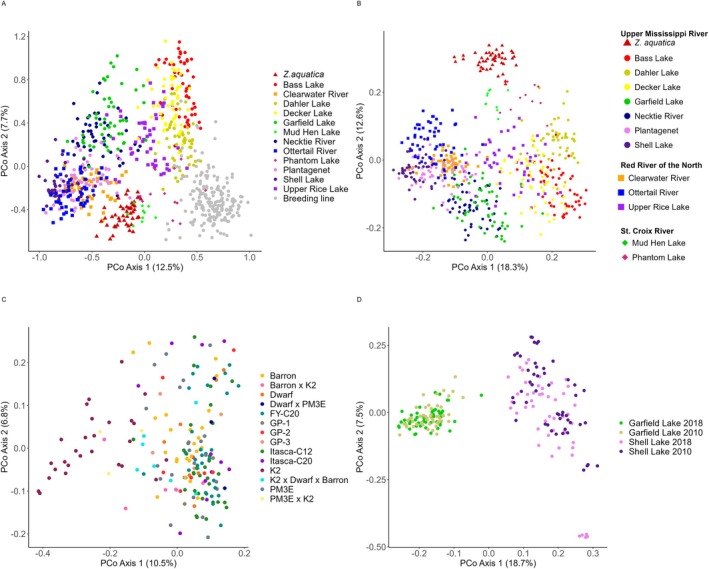
Principal coordinate analysis (PCoA) showing the differentiation of the 1st and 2nd principal coordinates of (A) the Natural Stand and Cultivated collections of Northern Wild Rice (NWR; *Zizania palustris* L.); (B) the Natural Stand collection; (C) the Cultivated collection; and (D) the Temporal collection.

To visualize the variation within the diversity collection, we first performed PCoA on both the Natural Stand and Cultivated collections together (Figure 2A). The first three principal coordinates explained 12.5%, 7.7%, and 7.3% of the variance, respectively (27.5% total). Within the first coordinate, samples were primarily split into two clusters, including a cluster of all *Z. aquatica* samples and the majority of Natural Stand samples, and a second cluster including all Cultivated samples and Bass and Decker Lake populations from the UMR watershed (Figure 2A). Upper Rice Lake genotypes appeared to blend between these two main groups but trended more heavily towards the group containing the UMR watershed genotypes. Within the first two principal coordinates, a large range of continuous variation can be seen, with samples primarily grouped by their germplasm type and geographic origin. Overall, these two principal coordinates failed to fully separate individual populations, though most *Z. aquatica* samples did separate from those of *Z. palustris* (Figure 2A). The Cultivated collection largely formed its own group with only a small number of samples overlapping with those from Bass, Upper Rice, Decker, Dahler, and Phantom (WI) Lakes. The third principal coordinate, in conjunction with coordinate 1, isolated *Z. aquatica* from all *Z. palustris* samples, Natural Stand and Cultivated collections, while maintaining the separation of the two main groups defined by coordinates 1 and 2 (Figure S2).

When analyzed separately from the Cultivated collection, the first two coordinates of the Natural Stand collection explained 19% and 13% of the variance, respectively (Figure 2B). The two main clusters were consistent with Figure 2A, as was Upper Rice Lake bridging the two clusters (Figure 2B), while *Z. aquatica* formed a more distinct cluster in principal coordinate 2. Natural Stand populations from the SCR fell between *Z. aquatica* and the rest of the MN *Z. palustris* populations, with only one *Z. palustris* sample from Dahler Lake overlapping with each of these groups. Other populations appear to cluster along a geographical gradient rather than by watershed. For example, the samples from Clearwater and Ottertail Rivers in the RRN, and Plantagenet and Shell Lakes in the UMR, which are located near one another, also grouped closely together (Figure 2B; Table S2). There was also a more defined population structure among UMR populations, with several smaller grouping patterns within the larger two clusters, including Necktie River with Garfield Lake, and Bass Lake with Dahler and Decker Lakes populations.

Compared to the Natural Stand collection, there was far less genetic structure evident among genotypes of the 4 cultivars and 10 breeding lines of the Cultivated collection (Figure 2A,C). When the Cultivated collection was analyzed alone, the first and second principal coordinates explained 10% and 7% of the variation, respectively (Figure 2C). The most distinct cluster within the Cultivated collection consisted of samples of “K2”, which is a cNWR cultivar released in 1972 that has been kept spatially isolated from other cNWR populations for at least the last 20+ years. While some grouping was present between individuals of a variety or breeding population, there was an overall lack of population structure among Cultivated materials.

To further explore the genetic relationships within the collection, neighbornet diagrams were generated for individual samples (Figure S3) as well as on a population basis (Figure S4). The results were largely consistent with the clustering observed in the PCoA (Figure 2A–C), wherein individuals grouped according to their population identity or sampling location. Notably, all individuals from Decker Lake, Clearwater River, Ottertail River, Shell Lake, Necktie River, Mud Hen Lake, and Lake Plantagenet clustered, as did 98% of *Z. aquatica* , Dahler Lake, and Cultivated individuals, and 96% of Bass Lake individuals (Figure S3). A total of 84% of individuals from Upper Rice Lake and Garfield Lake clustered, while the majority of remaining individuals grouped with a subset of individuals from Bass Lake (1 sample), Phantom Lake (Rogosin 1954), and the Cultivated collection (Elliott 1980).

Within both neighbor network diagrams, *Z. aquatica* was set as an outgroup (Figures S3 and S4). With the exception of a small number of samples, individuals grouped according to their location of origin, including the majority of Cultivated samples grouping together. The network separated further into several groupings, including Necktie (UMR) and Garfield (UMN) Lakes; Ottertail River (RRN) and Shell Lake (UMR); Clearwater River (RRN) and Lake Plantagenet (UMR); and Bass (UMR) and Decker (UMR) Lakes in the individual network, which also grouped with Dahler (UMR) Lake in the population network (Figures S3 and S4). Upper Rice Lake (UMR) does not cluster directly with any other populations but is most closely grouped with Decker and Bass Lakes (Figures S3 and S4). The Cultivated samples clustered most closely with other Cultivated samples, grouping between Dahler and Bass Lakes. Consistent with the PCoA, there was minimal structure within Cultivated samples. Reticulations were identified among these groupings, including with *Z. aquatica* (Figure S3).

**FIGURE 3:**
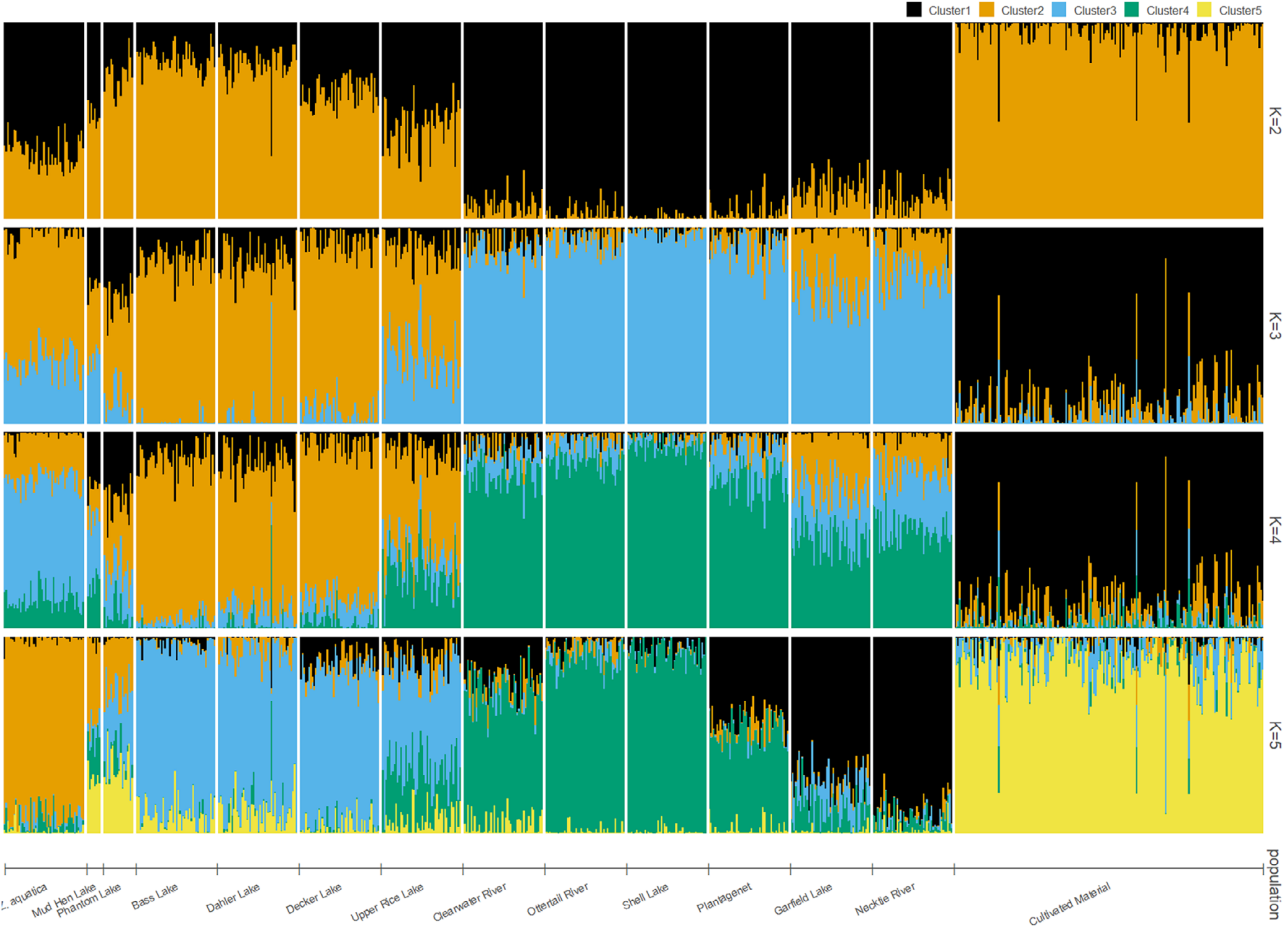
Population structure analysis of Northern Wild Rice (NWR; *Zizania palustris* L.) Natural Stand and Cultivated collections using the program STRUCTURE with 10,000 reps and a burn‐in length of 1000 for *K* = 2–5.

Using STRUCTURE analyses from *K* = 2–14, evidence of admixture can be seen across species, geographic origin, and germplasm type (Figure 3 and Figure S5), similar to the results of the PCoA and neighbor network analyses. Although there was a significant decrease in the DeltaK statistic between *K* = 2 and *K* = 3, the lowest value was found at *K* = 5 (Figure S6). Most notably, *Z. aquatica* separated from the Natural Stand populations for the first time at *K* = 5, while the Cultivated collection formed its own cluster and the Natural Stands grouped similarly to the PCoA plots and neighbor network diagrams (Figure 3). The vast majority of the collections showed limited admixture (< 1%) between different populations, with the exceptions of Upper Rice Lake and Phantom Lake. Upper Rice Lake showed heavy admixture with Decker Lake, Dahler Lake, Bass Lake, Ottertail River, and Shell Lake populations. Phantom Lake showed an average of 21.43% admixture with the Cultivated materials. Other population groupings were also informative; for example, at *K* = 3, we observed the Cultivated collection separate into its own cluster, while the other two clusters consisted of the Natural Stand populations and *Z. aquatica* (Figure 3).

Interesting patterns were found at higher *K* values as well. At *K* = 10, *Z. aquatica* and the Cultivated collection remained largely unchanged from *K* = 5 as did Bass Lake, Clearwater River, and Necktie River populations (Figure S5). Populations with high admixture including Phantom and Upper Rice Lakes also remained largely unchanged. However, the Mud Hen Lake population, which showed high admixture at *K* = 5, formed a unique cluster at *K* = 10, possibly owing to its distance from other sampling sites, as one of only two lakes collected in Wisconsin. Mud Hen Lake was further delineated at *K* = 14, splitting into two unique clusters. The results of *K* = 14 were otherwise consistent with *K* = 10 (Figure S5).

### 3.3 Analysis of Molecular Variance

Analysis of Molecular Variance (AMOVA) was conducted between several different groupings including: (1) Natural Stand populations vs. Natural Stand populations; (2) Cultivated lines vs. Cultivated lines; (3) Natural Stand populations vs. Cultivated lines; and (4) *Z. palustris* individuals versus *Z. aquatica* individuals. The AMOVA results revealed more variation within rather than between groups (Table 2). All the comparative groups identified that 3.37%–8.10% of the variation could be attributed to differences among the groups, rather than within. The highest and lowest variation among groups were identified in the Natural Stand vs. Natural Stand analysis (8.10%) and the Cultivated vs. Cultivated analysis (3.37%), respectively (Table 2).

**TABLE 2:** Analysis of Molecular Variance (AMOVA) of a Northern Wild Rice (NWR; *Zizania palustris* L.) diversity collection grouped by germplasm and species type based on 5955 single‐nucleotide polymorphism (SNP) markers generated via genotyping‐by‐sequencing (GBS).

Grouping	Source of variation	df	MS	Sigma	% of total	*p*
Natural stand versus Natural stand	Variation among populations	12	7440.64	133.71	8.10	0.001
	Variation within populations	567	1516.08	1516.08	91.90	0.001
	Total of variation	579	1638.87	1649.78	100.00	0.001
Cultivated versus Cultivated	Variation among populations	13	1592.63	18.31	1.33	0.001
	Variation within populations	172	1360.34	1360.34	98.67	0.001
	Total of variation	185	1376.66	1378.65	100.00	0.001
Natural stand versus Cultivated	Variation among populations	1	20,955.05	68.42	4.09	0.001
	Variation within populations	765	1604.18	1604.17	95.91	0.001
	Total of variation	766	1629.44	1672.60	100.00	0.001
*Z. palustris* versus *Z. aquatica*	Variation among populations	1	9517.98	86.37	5.05	0.001
	Variation within populations	578	1625.24	1625.23	94.95	0.001
	Total of variation	579	1638.87	1711.61	100.00	0.001

Abbreviations: df, degrees of freedom; MS, mean squares.

### 3.4 Gene Flow

**FIGURE 4:**
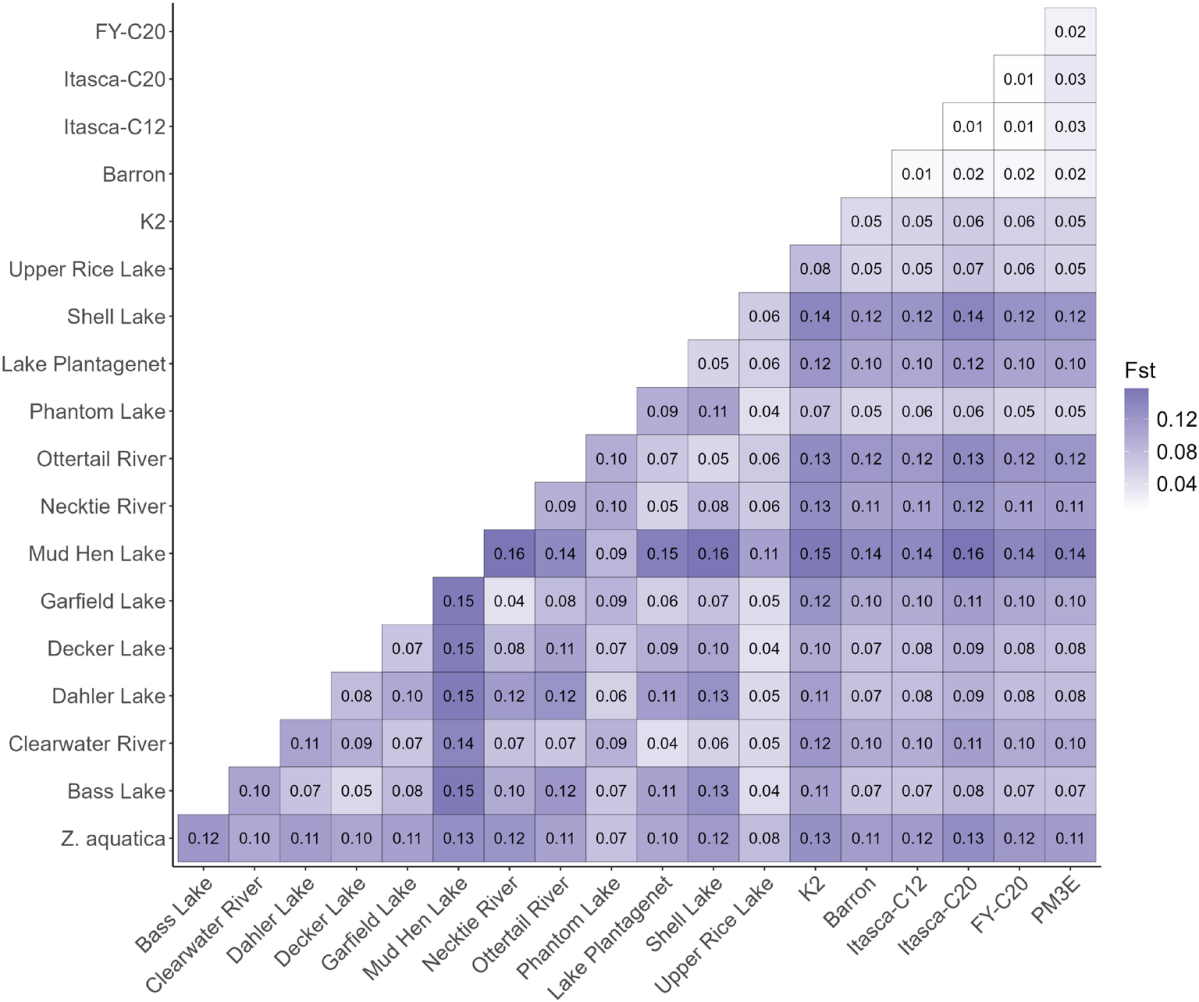
Fixation index (*F*_ST_) values derived using the weighted Weir and Cockerham method (Weir and Cockerham 1984) for a Natural Stand collection and a Cultivated collection of Northern Wild Rice (NWR; *Zizania palustris* L.).

*F*_ST_ values were calculated to compare the genetic differentiation within and among the Natural Stand and Cultivated collections (Figure 4). Overall, genetic differentiation between the Natural Stand populations was 0.09, with a range of 0.04–0.16. Pairwise comparisons that included Mud Hen Lake had the highest *F*_ST_ values, most above 0.13, indicating high differentiation from other Natural Stand populations. The Mud Hen Lake population was most similar to Phantom Lake (0.09) and Upper Rice Lake (0.01) populations. Overall, Necktie River and Garfield Lake (*F*_ST_ = 0.04) and Decker and Upper Rice Lakes (*F*_ST_ = 0.04) were the most similar populations. As seen with the results of the PCoA, Upper Rice Lake appeared similar to a number of other Natural Stand populations, including Bass, Decker, and Phantom Lake populations, which all had *F*_ST_ values below 0.05. Although most pairwise comparisons between *Z. aquatica* and the Natural Stand populations had *F*_ST_ values above 0.1, Upper Rice Lake and Phantom Lake values were slightly lower at *F*_ST_ = 0.08 and 0.07, respectively.

The pairwise comparisons between Cultivated populations had much lower *F*_ST_ values, with an average of 0.03 and a range of 0.01–0.06, indicating less differentiation than the Natural Stand populations. For pairwise comparisons between Natural Stand and Cultivated populations, the average *F*_ST_ value was 0.10, with a range of 0.05–0.16. Phantom lake showed the highest similarity to the Cultivated populations, with *F*_ST_ values below 0.06 for all comparisons except that with K2 (*F*_ST_ = 0.07). Mud Hen Lake comparisons with Cultivated populations had the highest *F*_ST_ values, all falling above 0.13, similar to its comparisons with other Natural Stand populations. *Z. aquatica* pairwise comparisons also had relatively high *F*_ST_ values, with a range of 0.11–0.13.

Migration rates between populations were also calculated to assess gene flow (Table S6). The rate of migration into the *Z. aquatica* outgroup was 9.2%, with 5.8% from Natural Stand populations and 3.4% from Cultivated populations, and it contributed 0.5%–1.1% of migration into other populations. Percents of migration into Natural Stands ranged from 8.7%–31.9%, with 0.48%–3.49% from Cultivated lines and 8.2%–19.5% from other Natural Stands. Although the overall migration levels into Natural Stand populations were low, there were higher levels for Plantagenet (31.9%) and Mud Hen Lake (20.7%). Migration into Cultivated lines ranged from 16.1%–32.2%, with 15.4%–31.1% from other Cultivated populations and 0.68%–1.8% from Natural Stands. The Itasca‐C12 and K2 populations had the lowest migration values (16.1% and 20.4%, respectively) of the Cultivated populations. However, there was an overall higher level of migration into Cultivated populations than Natural Stand populations, and the majority of the high migration values (> 10%) were found between Cultivated populations. In addition, Itasca‐C20 and Mud Hen Lake showed evidence of moderate migration (1%–10%) from most of the other populations, Natural Stand and Cultivated.

To further examine the basis for the observed genetic differentiation, we used a Mantel test to look for correlation between genetic and geographic distances of the Natural Stand collection. A positive correlation between the two was observed (Grombacher et al. 1997; *r* = 0.4011) and the best fit line yielded a regression equation of y=0.1+0.0002x (Figure S7a). An isolation by distance plot was also made for comparison, which also found a positive correlation (Grombacher et al. 1997; *r* = 0.2982) and had a regression equation of y=0.06+0.0002x (Figure S7b). Permutation tests showed that the observed results were unlikely to have occurred by chance (*p*‐value < 0.05; Figure S8). We were also interested in whether we could detect admixture between our Natural Stand and Cultivated collections. Using *D*‐statistics, we analyzed three groups including one Cultivated group, two Natural Stand groups based on PCoA and STRUCTURE analyses, along with *Z. aquatica* , which served as an outgroup, and found that there was no significant admixture between the groups (*Z* = 1.66, *p* = 0.098; Table S7).

### 3.5 The Temporal Collection

We examined the effect of time on the genetic diversity of two Natural Stand populations collected in 2010 and 2018, using the same approaches used to compare Natural Stand and Cultivated collections. The first principal coordinate of the PCoA for the Temporal collection separated Garfield and Shell Lakes, demonstrating population‐level differences. Meanwhile, the second coordinate, which explained 7% of the variation, was largely attributed to the temporal variation between Shell Lake samples collected in 2010 versus 2018 (Figure 2D). Within the first two principal components, the Garfield Lake population did not separate by time points. The Shell Lake population, on the contrary, demonstrated a wider range of variation in 2010 than it did in 2018. Additionally, a small unique cluster, not identified in 2010, was identified in 2018 (Figure 2D). Similar patterns were found in the UPGMA tree with monophyletic clades defined for both lakes (Figure S9). There was an even distribution of clustering patterns between Garfield Lake samples collected in 2010 and 2018. For Shell Lake, we observed one unique cluster consisting of 76% of the 2010 samples and 22% of the 2018 samples. The remaining samples showed minimal clustering with few differences between samples collected at different time points (Figure S9). The pairwise comparisons of each lake's genetic differentiation over time further substantiated these findings as the Garfield Lake 2010 versus 2018 comparison had a lower *F*_ST_ value of 0.0004 and Shell Lake in the same two time periods had a higher value of 0.0125 (Figure 4).

### 3.6 Scanning for Signatures of Selection

**FIGURE 5:**
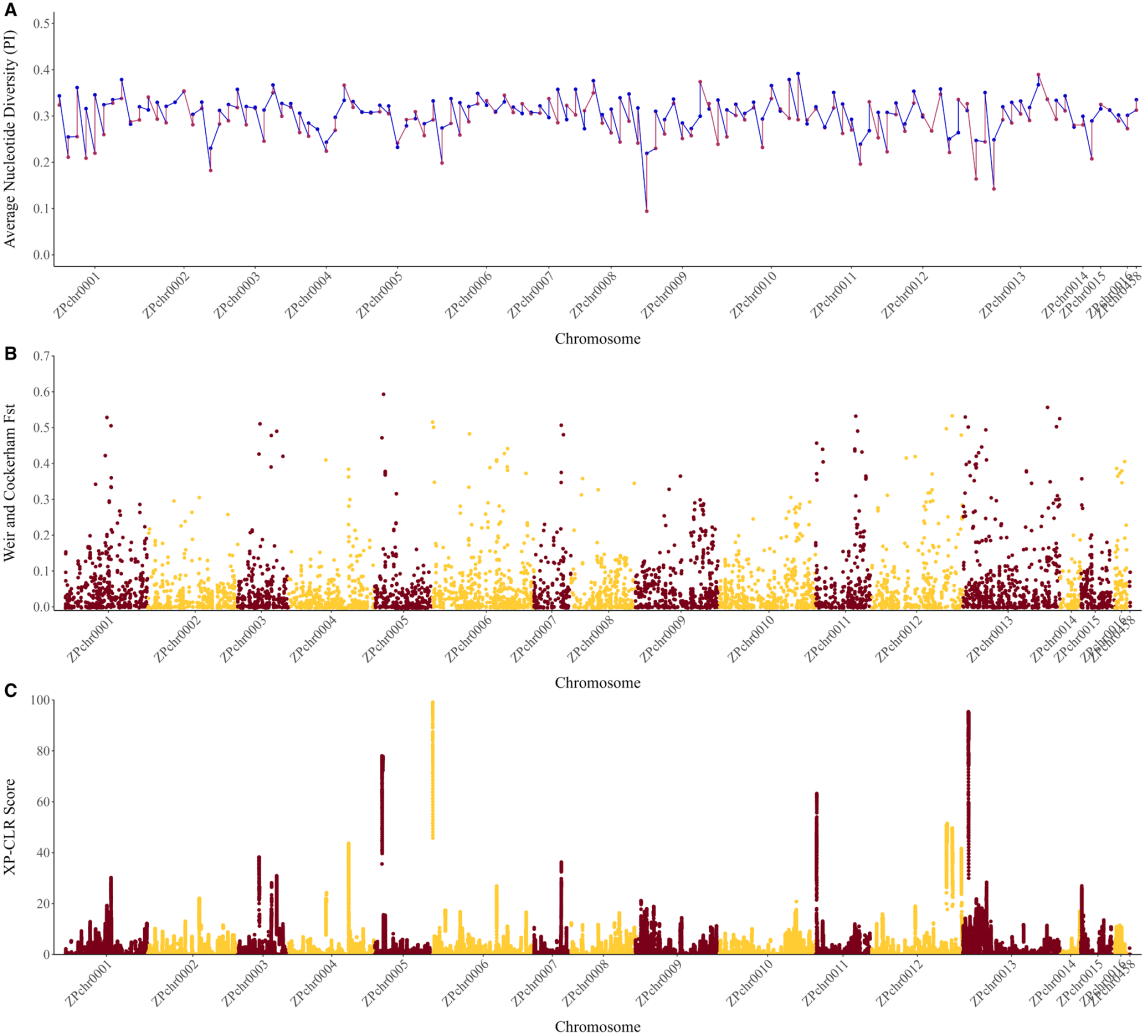
Genome‐wide scans of (A) nucleotide diversity (π; Nei 1987) of a Natural Stand collection (blue) and a Cultivated collection (red) of Northern Wild Rice (NWR; *Zizania palustris* L.); (B) Fixation index (*F*_ST_) values; and (C) XP‐CLR scores.

To identify genomic regions potentially subjected to selection or other genetic bottlenecks in cNWR, we calculated genome‐wide statistics for nucleotide diversity (π), *F*_ST_, and XP‐CLR tests (Figure 5). While we observed considerable similarity between π values of the Natural Stand and Cultivated collections across the genome (Figure 5A), we identified 25 bins with negative Tajima's *D* values in the Cultivated collection (Table S8). The most negative Tajima's *D* was −0.192 at ~50 Mb on ZPchr0009. Genome‐wide scans of *F*_ST_ values calculated between Natural Stand and Cultivated collections identified 166 individual pairwise comparisons above 0.10; 63 above 0.20; 24 above 0.30; 9 above 0.40; and 3 above 0.50 (Figure 5B). Genomic positions for these hits are listed in Table S8. The top 1% of *F*_ST_ scores were identified on ZPchr0007, ZPchr0009, and ZPchr0013. Finally, we identified 9 regions of the genome with XP‐CLR scores above 40 and 4 regions with scores above 60 (Figure 5C). The top 1% of these scores included 468 SNPs, including 141 hits on ZPchr0005 between 8.5 and 9.7 Mb, 112 hits on ZPchr0006 between 1.2 and 1.4 Mb, 22 hits on ZPchr0011 between 1.3 and 1.4 Mb, and 189 hits on ZPchr0013 between 6.2 and 6.8 Mb (Table S8). Allele frequencies of SNPs in these regions were all fixed in the Cultivated collection and ~0.50/0.50 in the Natural Stand collection.

No putative regions of interest were identified across all three tests. However, 11 5‐Mb regions with the top 1% of Tajima's *D* and *F*_ST_ scores were identified, including 2 regions on ZPchr0001, 2 regions on ZPchr0002, 2 on ZPchr0008, 2 regions on ZPchr0009, 1 on ZPchr0010, 1 on ZPchr0012, and 1 on ZPchr0015 (Table S8). Two regions, less than 1 Mb each, with overlapping top 1% of *F*_ST_ and Xp‐CLR scores were identified on ZPchr0011 and ZPchr0013.

## 4 Discussion

### 4.1 Assessment of Northern Wild Rice Via Genotyping‐by‐Sequencing

Cost‐effective sequencing technologies that are capable of generating robust sets of genome‐wide molecular markers, such as GBS, are providing researchers, especially those working with complex plant genomes and limited public resources, an avenue for rapid variant detection (Bredeson et al. 2016; Delfini et al. 2021; García‐Abadillo et al. 2024; Migicovsky et al. 2021; Tuttle et al. 2024). In this study, we developed a large genome‐wide SNP dataset, aligned to the NWR reference genome, to assess the relationship between natural and cultivated populations as well as to provide a basis for future breeding and conservation studies. However, we want to emphasize that this GBS approach, which is based on *Btg1* and *TaqI* restriction enzymes, may have introduced a bias in allele frequencies due to polymorphisms in restriction sites (Arnold et al. 2013), which could have led to slightly skewed inferences regarding the population genetics of NWR. Future studies could utilize other low‐depth next‐generation sequencing approaches, such as whole genome skim sequencing, which would provide a snapshot of the genome without reducing allelic representation.

### 4.2 Relationships Within the Zizania Genus

Species of *Zizania* are endemic to North America (Xu et al. 2008, 2010) and split from the Oryzinae subtribe 20–30 million years ago (MYA) (Haas et al. 2021; Guo et al. 2015; Tang et al. 2010). Following this split, the Zizaniinae subtribe is hypothesized to have experienced a radiation of speciation across North America and into eastern Asia as individuals made it over the Bering land bridge, leading to the speciation of *Zizania latifolia* (Xu et al. 2015). Comparison of the *Z. palustris* and *Z. latifolia* genomes suggests that the two species split 6–8 MYA (Haas et al. 2021). Extant North American species split 0.7–1.1 MYA; this split was likely precipitated by increases in the habitat range of the *Zizania* progenitor species as climatic conditions shifted over the last million years (Xu et al. 2010; Walker 2011). Evidence suggests that *Zizania texana* , an endangered species living in a small stretch of the San Marcos River Valley in the southern US, is a relic, isolated population of the ancestral *Z. palustris* species (Xu et al. 2015). However, the evolutionary relationship between *Z. aquatica* , a species found from the Great Lakes region to the east coast of the continental US, and *Z. palustris* is not well understood. This is likely due to their overlapping range and interspecific crossability (Xu et al. 2015; Duvall and Biesboer 1988). In this study, the neighbor network diagrams, STRUCTURE, and principal coordinate analyses showed moderate support for the separation of *Z. palustris* and *Z. aquatica* . *F*_ST_ values were primarily affected by geographic distance and highest within *Z. palustris* rather than between *Z. palustris* vs. *Z. aquatica* (Figure 4). This may be due to the limited sample size in this study, and more research is needed to resolve the complex relationship between these *Zizania* species.

### 4.3 Structure of Northern Wild Rice Populations is Tied to Geography and a Complex History of Ecosystem Management

Previous genetic analyses of wild populations of NWR have found limited gene flow between populations and a lack of data to support a correlation between population structure and geographical location (Lu et al. 2005; Kahler et al. 2014; Xu et al. 2015). In this study, we did not find evidence of significant gene flow among wild populations of NWR, with populations by and large clustering according to their lake or river of origin (Figures 2 and 3, Figures S3 and S4). This level of differentiation was also evidenced by the moderate *F*_ST_ values (0.05–0.15) (Wright 1965) found between Natural Stand populations (Figure 4). The majority of our analyses, including the Mantel test and isolation by distance (Figure S7), do suggest a geographic basis for population structure in NWR. For example, Garfield Lake and Necktie River, the two closest populations in our study, displayed a high level of similarity with one another (Figures 2B and 3). These similarities may be due to ecotypic or adaptive variation, reflecting localized adaptations to shared environmental conditions or selective pressures in their habitats (Kawecki and Ebert 2004) but more research into the local adaptations in NWR is needed. While the primary drivers of adaptation and gene flow in NWR are not well understood (Lu et al. 2005) found that the area and size of an NWR population, along with its degree of isolation, were major factors affecting the genetic variability and gene flow among the NWR populations tested (Lu et al. 2005). Additionally, recent pollen travel studies found that most pollen is dispersed within the first 7 m for *Z. palustris* (Gietzel et al. 2022) and 1.5 m for *Z. texana* (Oxley et al. 2008), limiting the likelihood of high levels of gene flow via wind‐pollination in the genus.

The historical management and development of lakes across NWR's natural range have also likely contributed to the population structure identified in this study. Efforts to establish new stands of NWR as well as to address declining population sizes have resulted in reseeding efforts across the species' natural range (Porter 2019; Brandes 2019). For example, Upper Rice Lake (RRN), which is known to have undergone extensive reseeding efforts since the 1930s (Dr. Kimball, *personal communication*), clustered primarily with several UMR populations while showing limited overlap with other RRN populations (Figures 2b and 3). Upper Rice Lake also showed heavy admixture with a number of lakes in STRUCTURE analyses (Figure 3). Taken together, these results suggest that human intervention may have influenced the genetic variability and population structure of the Upper Rice Lake population assessed in this study. Additionally, Phantom Lake of the SCR watershed displayed heavy admixture with Cultivated materials. These results were surprising as Phantom Lake is one of the most geographically distant Natural Stand populations from cNWR production in this study, and closer populations displayed little to no admixture with Cultivated materials (Figure 3). However, Phantom Lake is part of the Crex Meadows State Wildlife Area and was artificially created in the 1950s, when a series of levee systems were installed. NWR restoration in this area began in 1991, with 500 lbs. (227 kg) of seed sown over the course of 3 years (Thompson et al. 2010). We hypothesize that at least a portion of the seed utilized in these efforts came from cNWR production, further highlighting the complexity of population genetic studies in NWR, as well as the importance of documenting seed sources used in reseeding efforts. We also suggest that future reseeding efforts should not use Phantom Lake populations as a seed source based on the recommendations of the Great Lakes Indian Fish & Wildlife Commission (David et al. 2019).

The data presented here for 12 wild populations of NWR likely represents only a small fraction of the species' genetic diversity. However, even with our small sample size, we were able to identify unique genetic variation within many of the populations in the Natural Stand collection. This indicates that for conservation efforts, it is important to consider populations of NWR individually as they may harbor unique alleles and may be more or less adapted to environmental change. Further studies, using a broader range and more even distribution of sampling locations, will increase our knowledge about the population structure and genetic relationships between wild NWR populations and aid with decision making for future reseeding and other conservation‐based efforts.

### 4.4 Towards Comprehensive Monitoring of Northern Wild Rice

Comprehensive in situ species monitoring can provide a better understanding of the evolutionary change a species undergoes over time and help to identify targets for conservation efforts. However, like many species, NWR's natural range crosses international borders, including Indigenous sovereign nations, and state borders, with limited public availability of species' data and no comprehensive monitoring platform. The concept of Essential Biodiversity Variables (EBVs) was developed to address such issues and help build scalable monitoring systems for species to understand patterns and thus detect change in a timely manner (Hoban et al. 2022; Pereira et al. 2013). Genetic composition (i.e., genetic diversity) is one of six EBV classes identified as essential for monitoring and maintaining global biodiversity. The EBV concept also emphasizes repeated measures or collection of the same data at the same locations over time. While the main NWR collection evaluated in this study was assessed at a single time point, we also piloted a spatio‐temporal assessment of NWR by evaluating two populations, Garfield and Shell Lakes, in 2010 and 2018. Comparing these two time points, we identified limited change between samples from the Garfield Lake population collected in 2010 and 2018 (Figure 2D; *F*_ST_ = 0.0004), which may suggest stability within the population, possibly due to a large effective population size, limited genetic drift, or limited selection pressures during this time period. Conversely, we identified a reduction in diversity between samples from the Shell Lake population collected in 2018 compared to those collected in 2010 (Figure 2D; *F*_ST_ = 0.0125), suggesting moderate genetic differentiation in the population between the two time points. These results suggest that more frequent monitoring for at least some NWR populations (< 8 generations) are likely necessary to appropriately monitor changes. Periera et al. (2013), for example, suggested sampling every 1 to 5 years. While identifying the drivers of genetic change is outside the scope of this study, classifying other EBVs, such as the locations' ecologies and community compositions, could help to identify important indicators of these genetic changes, or lack thereof, across these time points. For example, NWR populations are known for their 3–5 year boom and bust cycles (Waheed 2021) and for their sensitivity to shoreline development and recreational activity (Hansen 2008), which Shell Lake has in the form of campgrounds and resorts. As NWR is an important target for conservation efforts, a holistic approach to monitoring the health of natural stands, such as the EBV concept lays out, would provide impactful data for resource managers and environmental agencies interested in the health and preservation of NWR populations across the species natural range.

### 4.5 Cultivated Northern Wild Rice is Distinct from Natural Stand Populations

Gene flow between domesticated crops and their wild counterparts can have significant impacts on both natural ecosystems and agricultural production systems. Genetic contamination, loss of identity and genetic diversity, and increased weediness are all potential consequences of gene flow (Gepts and Papa 2003). For these reasons, the extent of gene flow between crops and their wild cohorts has been evaluated in numerous species and found to be dependent on a variety of factors including, but not limited to, mating system (i.e., out‐crossing vs. selfing), the type and frequency of pollination (i.e., insect vs. wind), the selective (dis)advantage of particular domesticated traits (i.e., seed shattering resistance reducing seed dispersal), genetic drift, and genotype × environment interactions (Pereira et al. 2013; Waheed 2021; Gepts and Papa 2003; Jeong et al. 2019). Some studies, such as those in soybean ( *Glycine max* ), have identified limited gene flow, with domesticated and wild samples separating into monophyletic clades (Jeong et al. 2019; Li et al. 2010). Other studies have identified significant historical gene flow during domestication, such as Emmer wheat ( *Triticum dicoccon* ) (Luo et al. 2007), as well as ongoing gene flow between crop‐weed complexes, such as those in cowpea ( *Vigna unguiculata* (L.) Walp) (Coulibaly et al. 2002), pearl millet ( *Pennisetum glaucum* ) (Mariac et al. 2006), and species in the *Sorghum* genus (Arriola and Ellstrand 1996; Sagnard et al. 2011).

Given the out‐crossing nature of NWR and that cNWR production occurs within the centers of origin and diversity of *Z. palustris* , it is important to understand the extent of gene flow between cultivated and wild populations. This study found that Natural Stand and Cultivated collections are genetically distinct from one another (Figures 2A and 3; Figure S5), indicating minimal gene flow between these two groups and corroborating the results of previous diversity studies in NWR using different marker systems (Lu et al. 2005; Diller et al. 2018; Kahler et al. 2014). However, based on the 1st principal coordinate from Figure 2A, we identified more similarities between the Cultivated collection and Bass, Decker, and Dahler Lakes than other Natural Stand populations. These lakes are geographically close to the UMN cNWR paddy complex in Grand Rapids, MN, and could suggest gene flow. However, it is more likely that this is due to a shared ancestral relationship, as neither STRUCTURE analysis (Figure 3) nor *D*‐statistics (Table S7) suggest recent gene flow between the two populations. Additionally, the majority of migration between populations in this study occurred between two Cultivated populations or two Natural Stand populations, with Cultivated to Natural Stand or Natural Stand to Cultivated contributing less to the overall migration values (Table S6). Importantly, the cultivated germplasm in use today is all descended from natural stand samples originally collected from this geographical region within the UMR watershed. Cultivation and domestication of NWR began in Aitkin, MN, and several small enterprises likely gathered seeds from local populations to build their germplasm bases (Oelke et al. 1982).

### 4.6 Domestication and Stewardship of Cultivated Northern Wild Rice

As domestication is a process rather than a specific event, species exhibit varying levels of domestication (Zeder et al. 2006). In cereals and other major agricultural crops, seed retention and size, seed dormancy and germination, plant growth habit, and plant size are domestication traits commonly targeted for selection (Stalker et al. 2021). The presence of these common traits across multiple taxa is known as the domestication syndrome, which differentiates domesticated species from their wild counterparts. While many of today's largest agricultural commodity crops have undergone mass selection for thousands of years, the advent of new technologies, such as genomic sequencing, provide today's plant breeders with new opportunities for the rapid, targeted domestication of new crops (Zhang et al. 2021). Additionally, these technologies afford researchers the opportunity to study the domestication process in real time (Ekar et al. 2019).

To begin exploring the domestication process of cNWR, we evaluated changes in nucleotide diversity levels and allele frequency distributions between Natural Stand and cNWR populations using Tajima's *D*, *F*_ST_, and XP‐CLR tests. No significant overlap was identified between the three tests, suggesting there is limited evidence for selective sweeps in cNWR. However, two 1‐Mb regions on ZPchr0011 and ZPchr0013 had overlapping top 1% of *F*_ST_ and Xp‐CLR scores, suggesting there is some evidence of genetic changes in cNWR compared to the Natural Stands (Figure 5). A preliminary scan of genes in these two regions identified 5 putative genes whose functions in other species, mainly white rice, are related to drought and salt stresses as well as abscisic acid (ABA) signaling. These included a *60S ribosomal protein kinase 32‐like* gene (Ji et al. 2016); a *CBL‐interacting protein kinase 32‐like* gene (Hu et al. 2016); an E3 ubiquitin‐protein ligase RZFP34 isoform X2 (Ding et al. 2015; Shu and Yang 2017); and two copies of *ras‐related protein RABC2a* (Mérida‐García et al. 2020). Unlike wild populations of NWR, cNWR is grown in man‐made irrigated paddies, which are drained shortly after flowering (Principal Phenological Stage 6; Duquette and Kimball 2020) to allow for mechanical harvesting of the grain. Therefore, cNWR experiences conditions similar to upland crops, for which standing water is not available during the development of fruit, ripening, and senescence. These results may suggest that stress‐related genes, particularly drought‐related genes, were heavily selected for in cNWR germplasm to adapt to this drastic change in environmental conditions compared with its natural habitats.

As XP‐CLR is more robust than *F*_ST_ for identifying recent selection events (Chen et al. 2010), we looked at the two additional XP‐CLR regions that contained the top 1% of the statistic's empirical distribution, including a region on ZPchr0005 between 8.5 and 9.7 Mb and a region on ZPchr0006 between 1.2 and 1.4 Mb. Within these regions, we identified a *calcium‐dependent protein kinase family protein* associated with drought and salt tolerance in white rice (Campo et al. 2014; Wei et al. 2014); a *2*,*3‐bisphosphoglycerate‐independent phosphoglycerate mutase‐like* gene involved with chlorophyll synthesis and photosynthesis in white rice (Lin et al. 2019); a *CTD nuclear envelope phosphatase 1 homolog* associated with seed shattering resistance in white rice (Yang et al. 2010); a *KH domain‐containing protein SPIN1‐like* associated with flowering time in white rice (Vega‐Sánchez et al. 2008); and a *pentatricopeptide repeat‐containing protein At1g11900 isoform X1* associated with male sterility in *Petunia* (Bentolila et al. 2002). Two paralogs of *cytochrome P450 714D1‐like* were identified on both ZPchr0005 and ZPchr0006 regions of interest. In white rice, this gene is associated with seed dormancy and flowering time (He et al. 2022; Nakagawa et al. 2002). While not in the scope of our current study, we think these regions merit further investigation. Given the significance of NWR to a wide range of stakeholders, it is important to understand the potential impact of gene flow from cNWR to wild NWR populations. Therefore, while understanding the domestication process in cNWR is important for the plant breeding process, it can also be used to monitor the genetic diversity of natural stands, allowing for better stewardship of these vital populations.

Domestication indices that account for varying levels of domestication have been proposed for several species and typically include: the extent of phenotypic differentiation between the domesticated species and its wild counterparts; the length of a species' domestication history; whether major genetic changes to the domesticated species have been identified; whether the species has been adapted to agricultural settings through targeted breeding efforts; and the extent of the species' cultivation (Clement 1999; Dempewolf et al. 2008; Hammer and Khoshbakht 2015). Cultivated NWR is somewhat phenotypically distinct from wild NWR, mainly in its growth habit and seed retention characteristics, which have been made possible through breeding efforts. While the species has a short history of cultivation, its production has expanded to California, which is outside the species' natural range. For these reasons, we suggest that cNWR should be classified as semi‐domesticated.

## 5 Conclusions

Northern Wild Rice is a species with ecological, cultural, and economic importance to the Great Lakes region of North America. Results suggest that wild NWR populations are genetically distinct from each other, and their population structure is influenced by their geographic distribution and possibly human intervention, such as reseeding efforts. Based on the preliminary temporal data found in this study, we believe it would be beneficial to monitor for shifts in the genetic diversity of NWR populations across both temporal and geographical scales. We also found that wild and cultivated NWR are genetically distinct and that gene flow between the two groups is limited. Cultivated germplasm has little population structure and, relative to other commercial crops, appears to be only semi‐domesticated. Nevertheless, we found putative selection signals that may be associated with traits that are unique to cultivated NWR, including drought tolerance and the bottlebrush panicle type. As the plant breeding process continues, loci with heavy domestication signatures can be used to monitor gene flow between wild and cultivated populations of NWR to expand upon the current conservation and stewardship practices for wild populations.

## Author Contributions

**Lillian McGilp:** formal analysis (equal), software (equal), validation (equal), visualization (equal), writing – original draft (equal), writing – review and editing (equal). **Matthew W. Haas:** data curation (equal), formal analysis (equal), methodology (equal), visualization (supporting), writing – original draft (supporting). **Mingqin Shao:** conceptualization (equal), resources (equal). **Reneth Millas:** formal analysis (supporting). **Claudia Castell‐Miller:** conceptualization (supporting), writing – review and editing (supporting). **Anthony J. Kern:** conceptualization (equal), resources (equal), writing – review and editing (equal). **Laura M. Shannon:** formal analysis (equal), investigation (equal), project administration (equal), supervision (equal), writing – original draft (equal), writing – review and editing (equal). **Jennifer A. Kimball:** conceptualization (equal), funding acquisition (equal), investigation (equal), methodology (equal), project administration (equal), supervision (equal), writing – original draft (equal), writing – review and editing (equal).

## Conflicts of Interest

The authors declare no conflicts of interest.

## Supporting information


**Figure S1.** A county‐level map of Minnesota and western Wisconsin showing where leaf tissue samples of the Northern Wild Rice (NWR; *Zizania palustris* L.) diversity collection were collected and highlighting counties with significant production of cultivated NWR. Colors and shapes correspond to those featured in the principal component analysis (PCA) plots (Figure 2a,b).

**Figure S2.** Principal coordinate (PCo) analysis (PCoA) showing the differentiation of the 1st and 3rd PCos of the Natural Stand and Cultivated collections of Northern Wild Rice (NWR; *Zizania palustris* L.).

**Figure S3.** Individual level NeighborNet diagram of the Natural Stands and Cultivated collections of Northern Wild Rice (NWR; *Zizania palustris* L.).

**Figure S4.** Population level NeighborNet diagram of the Natural Stands and Cultivated collections of Northern Wild Rice (NWR; *Zizania palustris* L.).

**Figure S5.** Population structure analysis of Northern Wild Rice (NWR; *Zizania palustris* L.) Natural Stand and Cultivated collections using the program STRUCTURE with 10,000 reps and a burn‐in length of 1000 for *K* = 5, 10, and 14.

**Figure S6.** A plot from STRUCTURE HARVESTER performed with the Evanno method based on 5955 single‐nucleotide polymorphism (SNP) markers generated via genotyping‐by‐sequencing (GBS) using the diversity collection of Northern Wild Rice (NWR; *Zizania palustris* L.).

**Figure S7.** Mantel test plots showing the correlation between geographic distance (*x*‐axis) and (a) genetic distance (*y*‐axis) and (b). *F*st/(1‐*F*st) for a Natural Stand collection of Northern Wild Rice (NWR; *Zizania palustris* L.). The regression lines, *y* = 0.1 + 0.0002*x* and *y* = 0.06 + 0.0002*x*, respectively were also plotted.

**Figure S8.** A histogram of the frequency of simulated correlation tests resulting from permutation testing for the Mantel test analysis of the Natural Stand collection of Northern Wild Rice (NWR; *Zizania palustris* ). The black diamond with a vertical line beneath it shows the actual correlation value from the Mantel (Figure S4) test using real data. This signifies that results are unlikely to have been reached by chance.

**Figure S9.** Unweighted pair group method with arithmetic averaging (UPGMA) cluster analysis with bootstrapping of the Temporal collection of Northern Wild Rice (NWR; *Zizaniapalustris* L.).

**Table S1.** List of samples in the diversity collection of Northern Wild Rice (NWR; *Zizania palustris* L.) genotyped with 5955 single‐nucleotide polymorphism (SNP) markers generated via genotyping‐by‐sequencing (GBS). HUC 8 watershed designations include the Upper Mississippi River (UMR), Red River of the North (RRN), and St. Croix River (SCR) basins. Samples were collected in 2010 and 2018.
**Table S2.** Geographic distance (km) matrix of lakes and rivers where Northern Wild Rice (NWR; *Zizania palustris* L.) and *Zizania aquatica* leaf tissue samples were collected. HUC‐8‐based watershed designations for the Upper Mississippi River (UMR), Red River of the North (RRN), and St. Croix River (SCR) are included.
**Table S4.** Marker statistics including the transition/transversion (TsTv) ratios for 5955 single‐nucleotide polymorphism (SNP) markers generated via genotyping‐by‐sequencing (GBS) using the diversity collection of Northern Wild Rice (NWR; *Zizania palustris* L.).
**Table S5.** Polymorphic Information Content (PIC) values for 5955 single‐nucleotide polymorphism (SNP) markers generated via genotyping‐by‐sequencing (GBS) using the diversity collection of Northern Wild Rice (NWR; *Zizania palustris* L.).
**Table S7.** *D‐*statistics (ABBA‐BABA) results for a diversity collection of Northern Wild Rice (NWR; *Zizania palustris* L.).

**Table S3.** List of samples in the diversity collection of Northern Wild Rice (NWR; *Zizania palustris* L.) sorted according to the National Center for Biotechnology Information Short Read Archive (NCBI SRA) BioSample accession numbers. The BioProject ID for the collection is PRJNA774842.

**Table S6.** Estimates of migration rates from BayesAss3‐SNPs analysis for a Natural Stand collection and a Cultivated collection of Northern Wild Rice (NWR; *Zizania palustris* L.).

**Table S8.** Significant values for Tajima’s *D*, *F*_ST_, and XP‐CLR scores for a diversity collection of Northern Wild Rice (NWR; *Zizania palustris* L.) based on 5955 single‐nucleotide polymorphism (SNP) markers generated via genotyping‐by‐sequencing (GBS).

## Data Availability

All data generated from this project have been deposited at the NCBI Sequence Read Archive under BioProject PRJNA774842. All code for the analyses described can be found at https://github.com/UMNKimballLab/WildRiceGeneticDiversity2022. Data files associated with this project have also been submitted to the Data Repository for the University of Minnesota (DRUM) and can be accessed at https://doi.org/10.13020/TPV1‐8J41.
