# Supplementary figures and images for: Towards Stewardship of Wild Species and Their Domesticated Counterparts: A Case Study in Northern Wild Rice (Zizania palustris L.)
Source: Ecol Evol. 2025 Mar 13;15(3):e71033. doi: 10.1002/ece3.71033 (PMC11906255; doi:10.1002/ece3.71033)

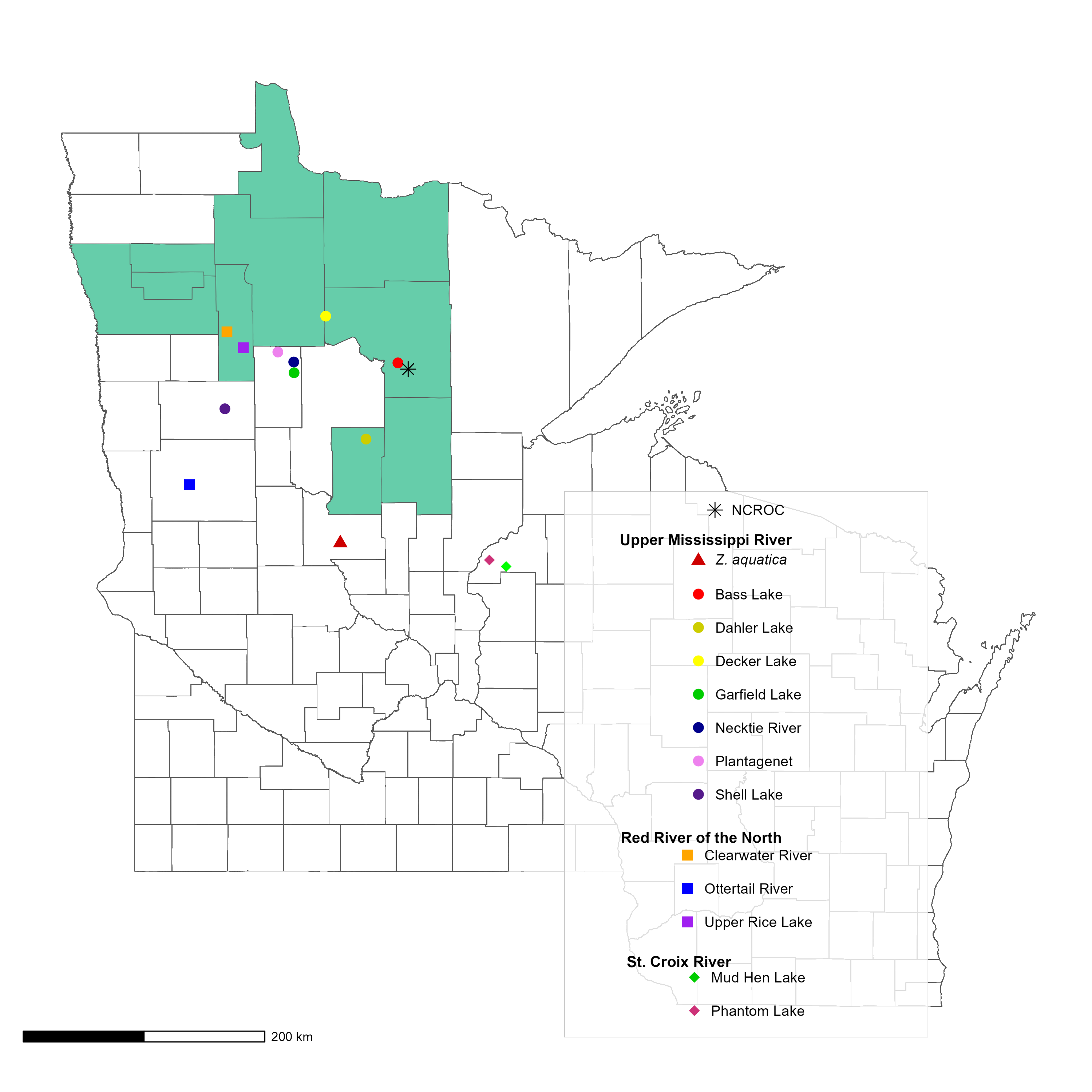

Supplement: Supplementary file 1 — Figure S1. A county‐level map of Minnesota and western Wisconsin showing where leaf tissue samples of the Northern Wild Rice (NWR; Zizania palustris L.) diversity collection were collected and highlighting counties with significant production of cultivated NWR. Colors and shapes correspond to those featured in the principal component analysis (PCA) plots (Figure 2a,b). [file ECE3-15-e71033-s011.png]

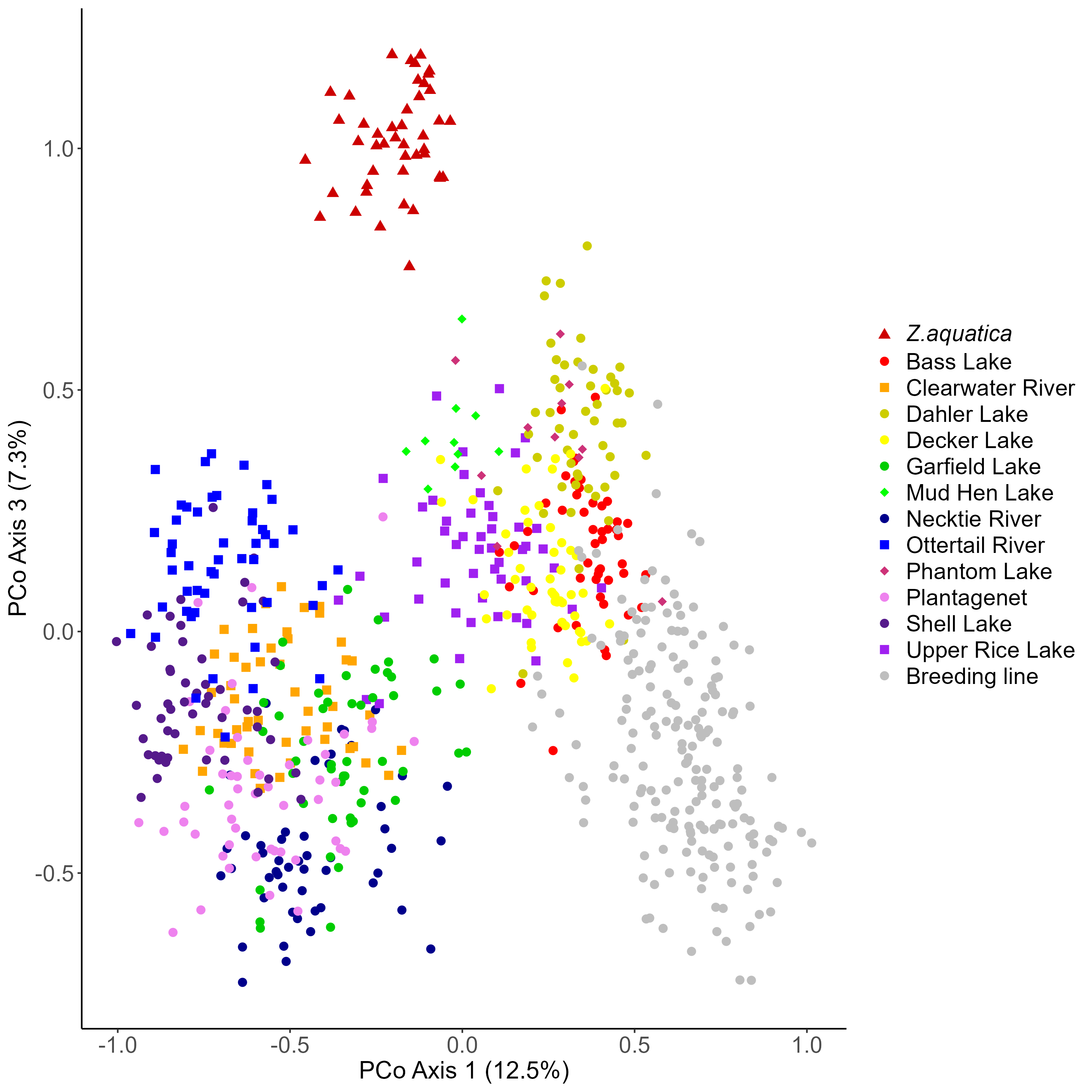

Supplement: Supplementary file 2 — Figure S2. Principal coordinate (PCo) analysis (PCoA) showing the differentiation of the 1st and 3rd PCos of the Natural Stand and Cultivated collections of Northern Wild Rice (NWR; Zizania palustris L.). [file ECE3-15-e71033-s005.png]

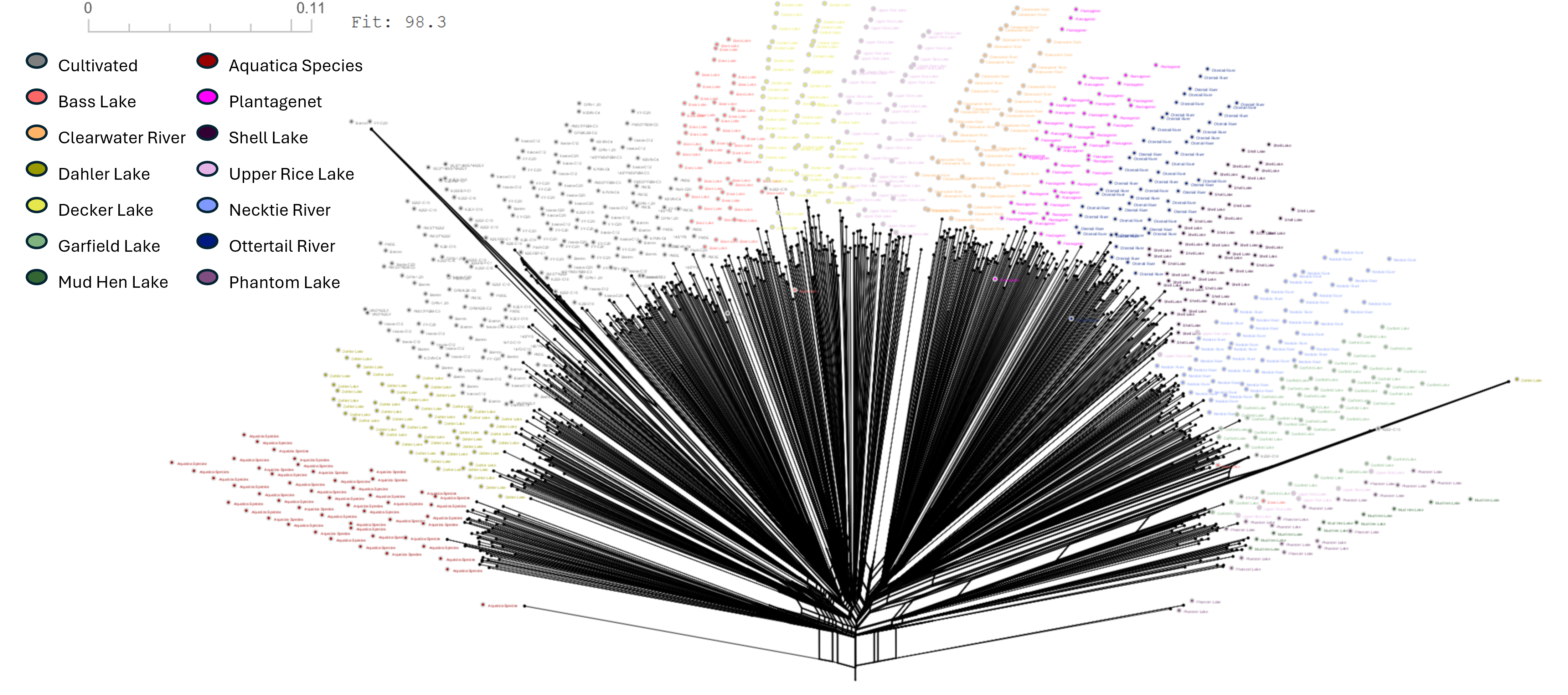

Supplement: Supplementary file 3 — Figure S3. Individual level NeighborNet diagram of the Natural Stands and Cultivated collections of Northern Wild Rice (NWR; Zizania palustris L.). [file ECE3-15-e71033-s003.png]

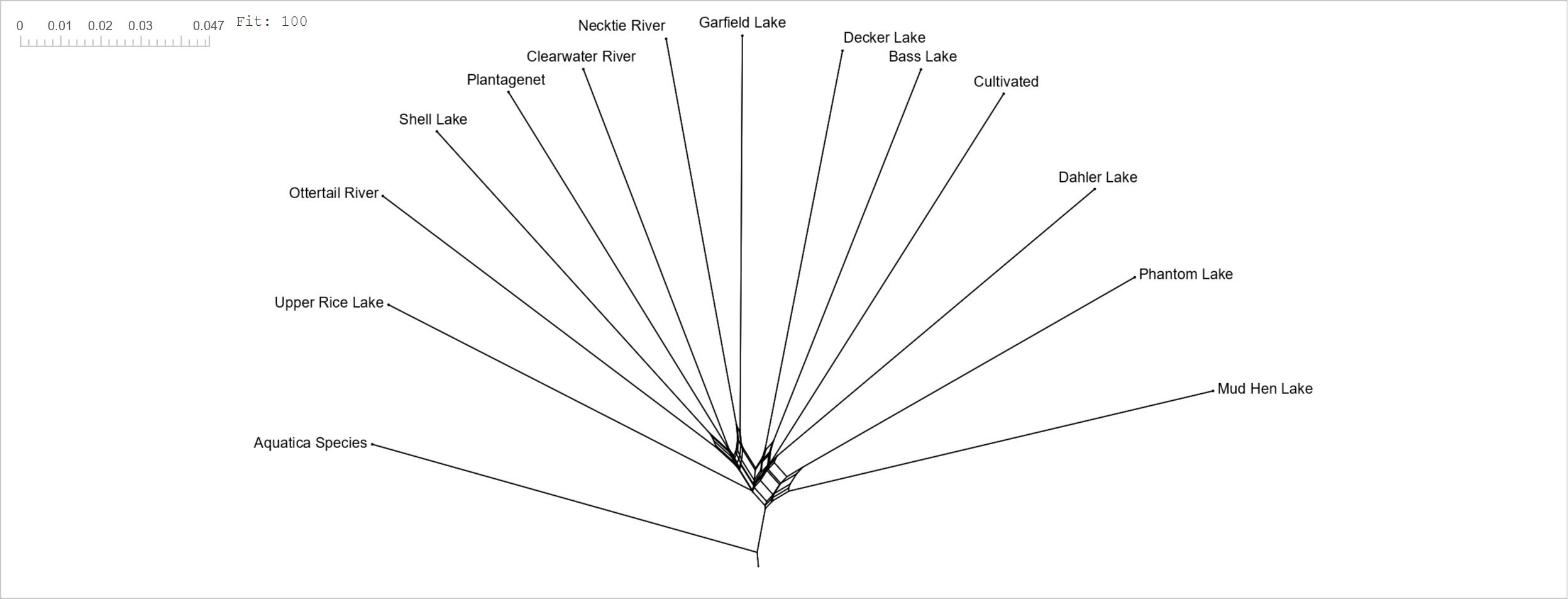

Supplement: Supplementary file 4 — Figure S4. Population level NeighborNet diagram of the Natural Stands and Cultivated collections of Northern Wild Rice (NWR; Zizania palustris L.). [file ECE3-15-e71033-s002.png]

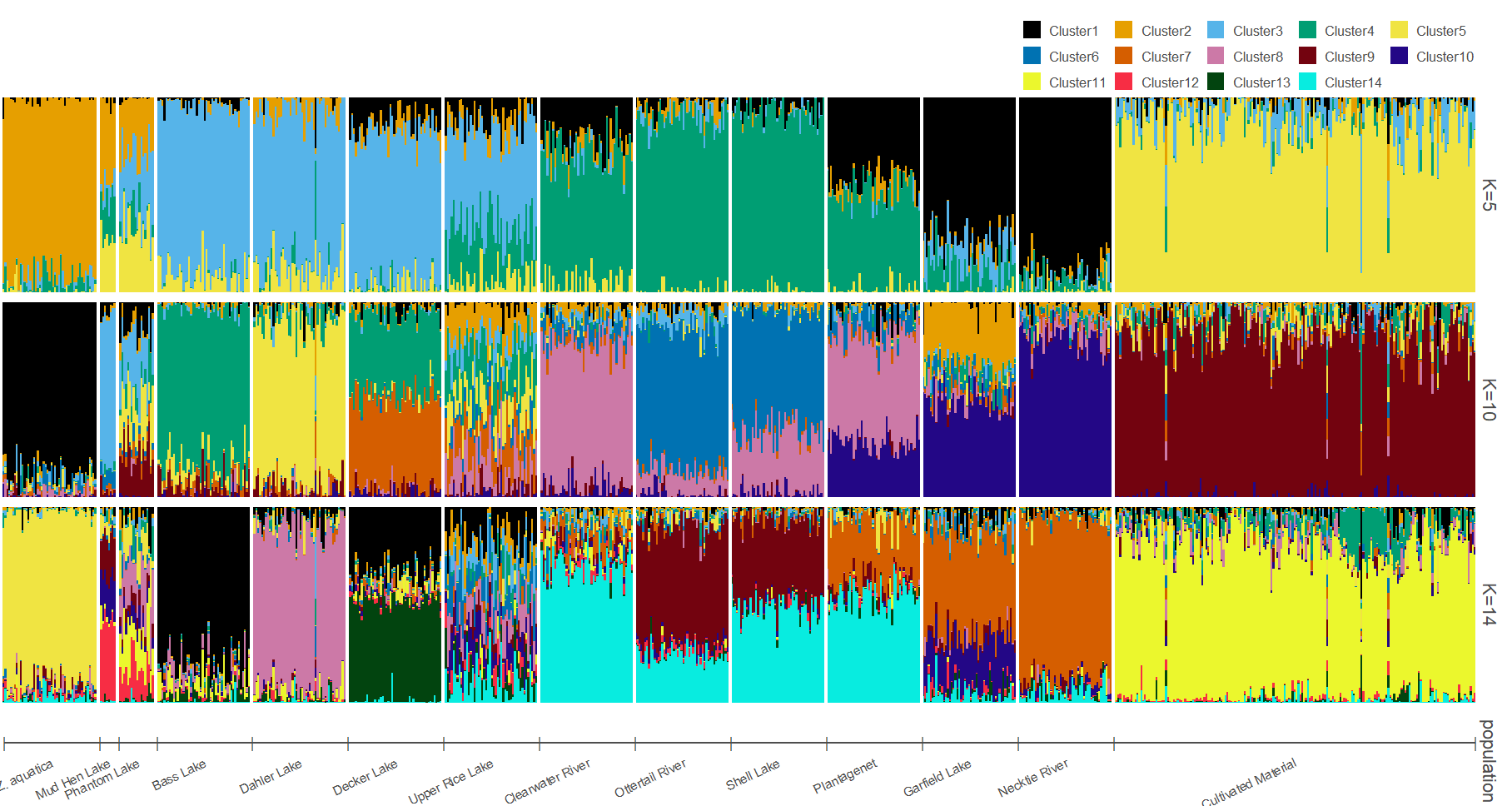

Supplement: Supplementary file 5 — Figure S5. Population structure analysis of Northern Wild Rice (NWR; Zizania palustris L.) Natural Stand and Cultivated collections using the program STRUCTURE with 10,000 reps and a burn‐in length of 1000 for K = 5, 10, and 14. [file ECE3-15-e71033-s009.png]

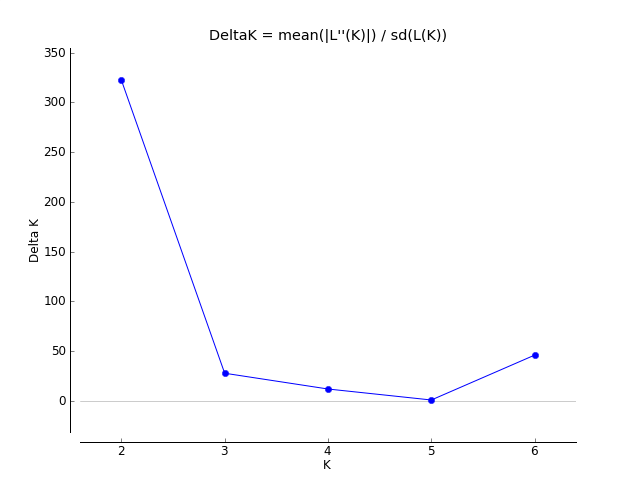

Supplement: Supplementary file 6 — Figure S6. A plot from STRUCTURE HARVESTER performed with the Evanno method based on 5955 single‐nucleotide polymorphism (SNP) markers generated via genotyping‐by‐sequencing (GBS) using the diversity collection of Northern Wild Rice (NWR; Zizania palustris L.). [file ECE3-15-e71033-s013.png]

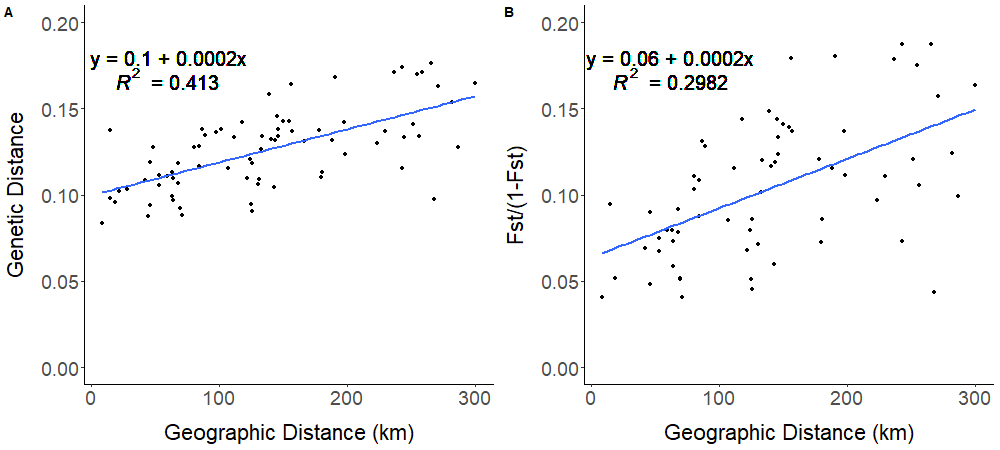

Supplement: Supplementary file 7 — Figure S7. Mantel test plots showing the correlation between geographic distance (x‐axis) and (a) genetic distance (y‐axis) and (b). Fst/(1‐Fst) for a Natural Stand collection of Northern Wild Rice (NWR; Zizania palustris L.). The regression lines, y = 0.1 + 0.0002x and y = 0.06 + 0.0002x, respectively were also plotted. [file ECE3-15-e71033-s001.png]

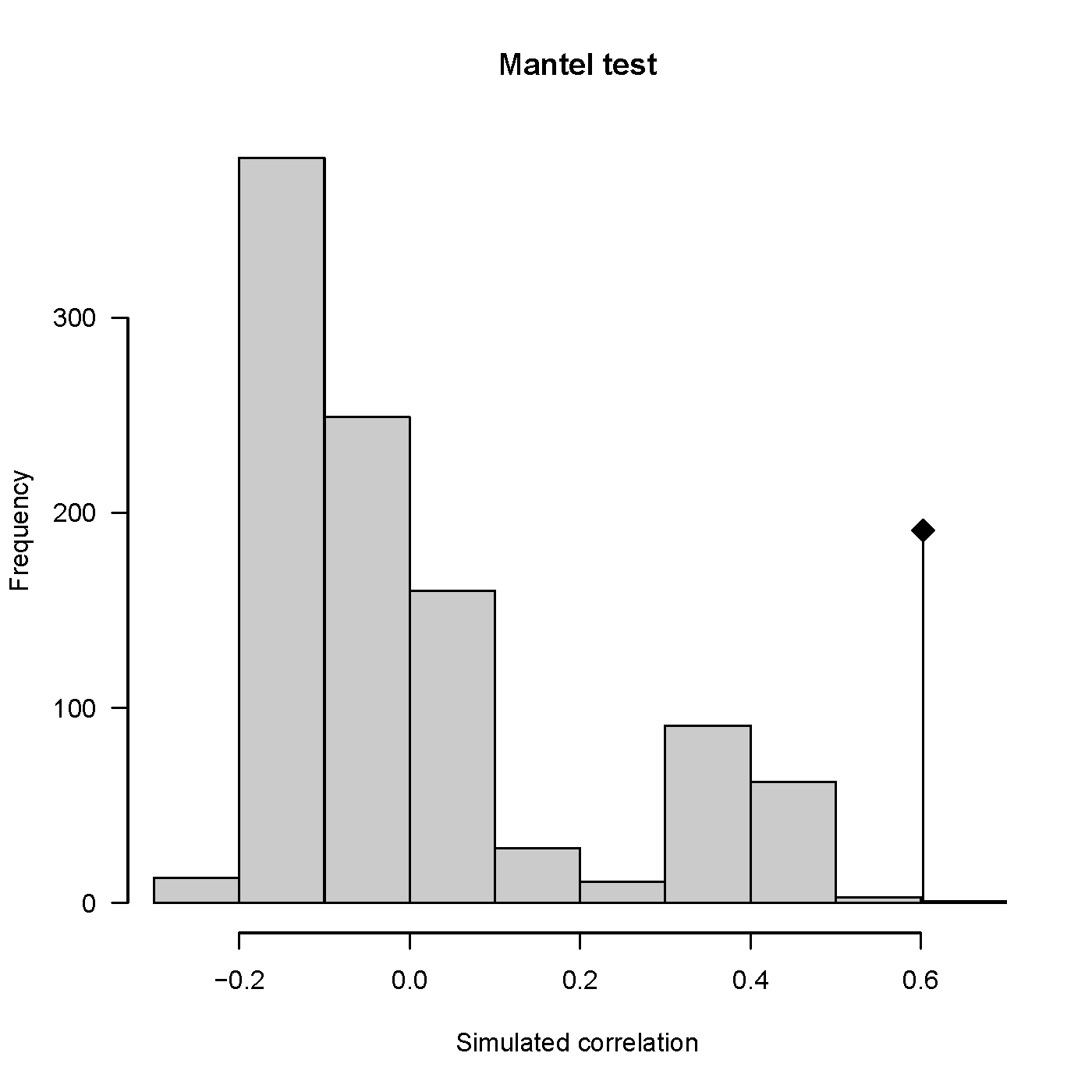

Supplement: Supplementary file 8 — Figure S8. A histogram of the frequency of simulated correlation tests resulting from permutation testing for the Mantel test analysis of the Natural Stand collection of Northern Wild Rice (NWR; Zizania palustris ). The black diamond with a vertical line beneath it shows the actual correlation value from the Mantel (Figure S4) test using real data. This signifies that results are unlikely to have been reached by chance. [file ECE3-15-e71033-s007.png]

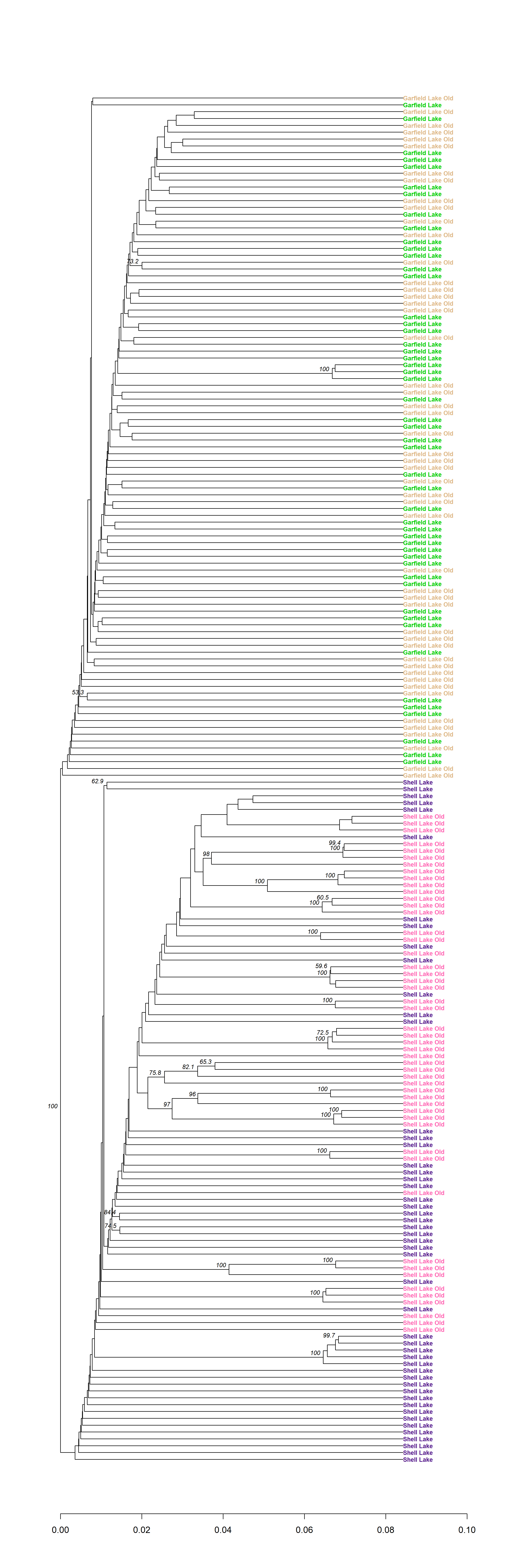

Supplement: Supplementary file 9 — Figure S9. Unweighted pair group method with arithmetic averaging (UPGMA) cluster analysis with bootstrapping of the Temporal collection of Northern Wild Rice (NWR; Zizaniapalustris L.). [file ECE3-15-e71033-s006.png]
