# Supplementary material for: Towards Stewardship of Wild Species and Their Domesticated Counterparts: A Case Study in Northern Wild Rice (Zizania palustris L.)
Source: Ecol Evol. 2025 Mar 13;15(3):e71033. doi: 10.1002/ece3.71033 (PMC11906255; doi:10.1002/ece3.71033)
Supplement: Supplementary file 10 — Table S1. List of samples in the diversity collection of Northern Wild Rice (NWR; Zizania palustris L.) genotyped with 5955 single‐nucleotide polymorphism (SNP) markers generated via genotyping‐by‐sequencing (GBS). HUC 8 watershed designations include the Upper Mississippi River (UMR), Red River of the North (RRN), and St. Croix River (SCR) basins. Samples were collected in 2010 and 2018. Table S2. Geographic distance (km) matrix of lakes and rivers where Northern Wild Rice (NWR; Zizania palustris L.) and Zizania aquatica leaf tissue samples were collected. HUC‐8‐based watershed designations for the Upper Mississippi River (UMR), Red River of the North (RRN), and St. Croix River (SCR) are included. Table S4. Marker statistics including the transition/transversion (TsTv) ratios for 5955 single‐nucleotide polymorphism (SNP) markers generated via genotyping‐by‐sequencing (GBS) using the diversity collection of Northern Wild Rice (NWR; Zizania palustris L.). Table S5. Polymorphic Information Content (PIC) values for 5955 single‐nucleotide polymorphism (SNP) markers generated via genotyping‐by‐sequencing (GBS) using the diversity collection of Northern Wild Rice (NWR; Zizania palustris L.). Table S7. D‐statistics (ABBA‐BABA) results for a diversity collection of Northern Wild Rice (NWR; Zizania palustris L.). [file ECE3-15-e71033-s008.docx]

**Table S1.** List of samples in the diversity collection of Northern Wild Rice (NWR; *Zizania palustris* L.) genotyped with 5,955 single nucleotide polymorphism (SNP) markers generated via genotyping-by-sequencing (GBS). HUC 8 watershed designations include Upper Mississippi River (UMR), Red River of the North (RRN), and St. Croix River (SCR) basins. Samples were collected in 2010 and 2018.

| **Sample identification** | **Number of individuals/population** | **Collection Type** | **GPS Coordinates**  **Latitud Longitud** | **Watershed** |
| --- | --- | --- | --- | --- |
| Bass Lake | 50 | Natural Stand | 47.28716 -93.63132 | UMR |
| Clearwater River | 50 | Natural Stand | 47.51838 -95.46291 | RRN |
| Dahler Lake | 50 | Natural Stand | 46.71888 -93.97281 | UMR |
| Decker Lake | 50 | Natural Stand | 47.63515 -94.40492 | UMR |
| Garfield Lake | 50 | Natural Stand/Temporal | 47.21361 -94.74380 | UMR |
| Mud Hen Lake | 10 | Natural Stand | 45.77005 -92.47173 | SCR |
| Necktie River | 50 | Natural Stand | 47.29363 -94.74724 | UMR |
| Ottertail River | 50 | Natural Stand | 46.38005 -95.86430 | RRN |
| Phantom Lake | 20 | Natural Stand | 45.81839 -92.65074 | SCR |
| Lake Plantagenet | 50 | Natural Stand | 47.36758 -94.91755 | UMR |
| Shell Lake | 50 | Natural Stand/Temporal | 46.94510 -95.48486 | UMR |
| Upper Rice Lake | 50 | Natural Stand | 47.40030 -95.28659 | RRN |
| *Zizania aquatica* | 50 | Natural Stand | 45.94473 -94.24821 | UMR |
| Garfield Lake 2010 | 50 | Temporal | 47.21361 -94.74380 | UMR |
| Shell Lake 2010 | 50 | Temporal | 46.94510 -95.48486 | UMR |
| Dwarf | 7 | Cultivated | n/a | n/a |
| Dwarf x PM3E | 3 | Cultivated | n/a | n/a |
| Barron | 25 | Cultivated | n/a | n/a |
| Itasca-C12 | 46 | Cultivated | n/a | n/a |
| Itasca-C20 | 11 | Cultivated | n/a | n/a |
| FY-C20 | 29 | Cultivated | n/a | n/a |
| GP-1 | 3 | Cultivated | n/a | n/a |
| GP-2 | 8 | Cultivated | n/a | n/a |
| K2 | 29 | Cultivated | n/a | n/a |
| K2 x Dwarf x Barron | 9 | Cultivated | n/a | n/a |
| GP-3 | 4 | Cultivated | n/a | n/a |
| PM3E x K2 | 3 | Cultivated | n/a | n/a |
| PM3E | 23 | Cultivated | n/a | n/a |
| Barron x K2 | 6 | Cultivated | n/a | n/a |

**Table S2.** Geographic distance (km) matrix of lakes and rivers where Northern Wild Rice (NWR; *Zizania palustris* L.) and *Zizania aquatica* leaf tissue samples were collected. HUC-8-based watershed designations for the Upper Mississippi River (UMR), Red River of the North (RRN), and St. Croix River (SCR) are included.

| Natural Population (Watershed) | *Z. aquatica* | Bass Lake | Clearwater River | Dahler Lake | Decker Lake | Garfield Lake | Mud Hen Lake | Necktie River | Ottertail Lake | Phantom Lake | Lake Plantagenet | Shell Lake | Upper Rice Lake |
| --- | --- | --- | --- | --- | --- | --- | --- | --- | --- | --- | --- | --- | --- |
| *Z. aquatica* (UMR) |  | 156.7 | 198.2 | 88.7 | 188.6 | 146.3 | 139.1 | 154.9 | 133.7 | 124.6 | 166.4 | 146.3 | 180.4 |
| Bass Lake (UMR) | 156.7 |  | 140.4 | 68.4 | 69.9 | 84.5 | 190.8 | 84.3 | 197.8 | 179.9 | 97.5 | 145.5 | 125.5 |
| Clearwater River (RRN) | 198.2 | 140.4 |  | 143.7 | 80.5 | 64.0 | 300.2 | 59.4 | 130.3 | 286.3 | 44.4 | 63.8 | 18.7 |
| Dahler Lake (UMR) | 88.7 | 68.4 | 143.7 |  | 107.1 | 80.4 | 156.6 | 86.9 | 149.6 | 142.8 | 101.7 | 117.9 | 125.2 |
| Decker Lake (UMR) | 188.6 | 69.9 | 80.5 | 107.1 |  | 53.4 | 254.7 | 45.9 | 178.3 | 242.5 | 48.7 | 112.0 | 71.3 |
| Garfield Lake (UMR) | 146.3 | 84.5 | 64.0 | 80.4 | 53.4 |  | 236.9 | 8.9 | 126.1 | 223.2 | 21.6 | 63.6 | 45.9 |
| Mud Hen Lake (SCR) | 139.1 | 190.8 | 300.2 | 156.6 | 254.7 | 236.9 |  | 243.2 | 270.6 | 14.9 | 258.2 | 265.9 | 281.6 |
| Necktie River (UMR) | 154.9 | 84.3 | 59.4 | 86.9 | 45.9 | 8.9 | 243.2 |  | 132.6 | 229.6 | 15.3 | 68.0 | 42.4 |
| Ottertail Lake (RRN) | 133.7 | 197.8 | 130.3 | 149.6 | 178.3 | 126.1 | 270.6 | 132.6 |  | 255.8 | 131.4 | 69.3 | 121.8 |
| Phantom Lake (SCR) | 124.6 | 179.9 | 286.3 | 142.8 | 242.5 | 223.2 | 14.9 | 229.6 | 255.8 |  | 244.5 | 251.2 | 267.6 |
| Lake Plantagenet (UMR) | 166.4 | 97.5 | 44.4 | 101.7 | 48.7 | 21.6 | 258.2 | 15.3 | 131.4 | 244.5 |  | 63.7 | 28.1 |
| Shell Lake (UMR) | 146.3 | 145.5 | 63.8 | 117.9 | 112.0 | 63.6 | 265.9 | 68.0 | 69.3 | 251.2 | 63.7 |  | 52.8 |
| Upper Rice Lake (RRN) | 180.4 | 125.5 | 18.7 | 125.2 | 71.3 | 45.9 | 281.6 | 42.4 | 121.8 | 267.6 | 28.1 | 52.8 |  |

**Table S3.** List of samples in the diversity collection of Northern Wild Rice (NWR; *Zizania palustris* L.) sorted according to the National Center for Biotechnology Information Short Read Archive (NCBI SRA) BioSample accession numbers. The BioProject ID for the collection is PRJNA774842.

*See External Excel File ‘Table S3’*

**Table S4.** Marker statistics including the-transition/transversion (TsTv) ratios for 5,955 single nucleotide polymorphism (SNP) markers generated via genotyping-by-sequencing (GBS) using the diversity collection of Northern Wild Rice (NWR; *Zizania palustris* L.)

|  | **ZPchr0001** | **ZPchr0002** | **ZPchr0003** | **ZPchr0004** | **ZPchr0005** | **ZPchr0006** | **ZPchr0007** | **ZPchr0008** | **ZPchr0009** |
| --- | --- | --- | --- | --- | --- | --- | --- | --- | --- |
| Scaffold (bp) | 95,470,783 | 103,377,072 | 58,865,324 | 98,769,966 | 66,616,710 | 118,006,097 | 42,614,780 | 75,690,682 | 95,187,837 |
| SNP # | 483 | 435 | 302 | 491 | 350 | 598 | 181 | 367 | 481 |
| SNPs/Mb | 5.06 | 4.21 | 5.13 | 4.97 | 5.25 | 5.07 | 4.25 | 4.85 | 5.05 |
| A/C | 40 | 33 | 30 | 38 | 17 | 60 | 23 | 29 | 44 |
| A/G | 169 | 171 | 108 | 205 | 147 | 197 | 57 | 128 | 181 |
| A/T | 22 | 16 | 12 | 18 | 17 | 26 | 10 | 13 | 16 |
| C/G | 19 | 19 | 17 | 19 | 13 | 34 | 17 | 16 | 17 |
| C/T | 197 | 174 | 109 | 178 | 131 | 233 | 58 | 152 | 196 |
| G/T | 36 | 22 | 26 | 33 | 25 | 48 | 16 | 29 | 27 |
| Ts | 366 | 345 | 217 | 383 | 278 | 430 | 115 | 280 | 377 |
| Tv | 117 | 90 | 85 | 108 | 72 | 168 | 66 | 87 | 104 |
| TsTv ratio | 3.13 | 3.83 | 2.55 | 3.55 | 3.86 | 2.56 | 1.74 | 3.22 | 3.63 |
|  | **ZPchr0010** | **ZPchr0011** | **ZPchr0012** | **ZPchr0013** | **ZPchr0014** | **ZPchr0015** | **ZPchr0016** | **ZPchr0458** | **Genome-wide** |
| Scaffold (bp) | 111,429,323 | 63,217,741 | 105,879,435 | 111,260,184 | 24,058,870 | 39,125,943 | 13,817,767 | 4,333,358 | 1,227,721,872 |
| SNP # | 568 | 285 | 410 | 583 | 91 | 237 | 88 | 5 | 5955 |
| SNPs/Mb | 5.10 | 4.51 | 3.87 | 5.24 | 3.78 | 6.06 | 6.37 | 1.15 | 4.85 |
| A/C | 49 | 21 | 25 | 43 | 7 | 15 | 6 | 0 | 480 |
| A/G | 213 | 109 | 149 | 234 | 30 | 92 | 40 | 2 | 2232 |
| A/T | 25 | 18 | 21 | 23 | 3 | 9 | 1 | 1 | 251 |
| C/G | 31 | 8 | 28 | 28 | 4 | 10 | 4 | 0 | 284 |
| C/T | 210 | 114 | 154 | 212 | 39 | 98 | 31 | 1 | 2287 |
| G/T | 40 | 15 | 33 | 43 | 8 | 13 | 6 | 1 | 421 |
| Ts | 423 | 223 | 303 | 446 | 69 | 190 | 71 | 3 | 4519 |
| Tv | 145 | 62 | 107 | 137 | 22 | 47 | 17 | 2 | 1436 |
| TsTv ratio | 2.92 | 3.60 | 2.83 | 3.26 | 3.14 | 4.04 | 4.18 | 1.50 | 3.15 |

**Table S5.** Polymorphic Information Content (PIC) values for 5,955 single nucleotide polymorphism (SNP) markers generated via genotyping-by-sequencing (GBS) using the diversity collection of Northern Wild Rice (NWR; *Zizania palustris* L.)

|  | Mean | Median | Minimum | Maximum | Standard Deviation |
| --- | --- | --- | --- | --- | --- |
| Barron | 0.1649 | 0.1557 | 0.0216 | 0.3125 | 0.1043 |
| FY-C20 | 0.1639 | 0.1557 | 0.0199 | 0.3125 | 0.1052 |
| Itasca-C12 | 0.1531 | 0.1379 | 0.0166 | 0.3125 | 0.1064 |
| Itasca-C20 | 0.1840 | 0.1829 | 0.0449 | 0.3125 | 0.0981 |
| K2 | 0.1803 | 0.1829 | 0.0261 | 0.3125 | 0.1053 |
| PM3E | 0.1774 | 0.1829 | 0.0292 | 0.3125 | 0.1041 |
| Bass Lake | 0.1484 | 0.1287 | 0.0010 | 0.3125 | 0.1093 |
| Clearwater River | 0.1523 | 0.1369 | 0.0113 | 0.3125 | 0.1084 |
| Dahler Lake | 0.1541 | 0.1373 | 0.0142 | 0.3125 | 0.1079 |
| Decker Lake | 0.1540 | 0.1395 | 0.0091 | 0.3125 | 0.1111 |
| Garfield Lake | 0.1471 | 0.1263 | 0.0091 | 0.3125 | 0.1090 |
| Mud Hen Lake | 0.1924 | 0.1999 | 0.0493 | 0.3125 | 0.0982 |
| Necktie River | 0.1500 | 0.1260 | 0.0113 | 0.3125 | 0.1108 |
| Ottertail River | 0.1528 | 0.1353 | 0.0010 | 0.3125 | 0.1104 |
| Phantom Lake | 0.1637 | 0.1702 | 0.0248 | 0.3125 | 0.1049 |
| (Lake) Plantagenet | 0.1534 | 0.1353 | 0.0010 | 0.3125 | 0.1105 |
| Shell Lake | 0.1477 | 0.1221 | 0.0010 | 0.3125 | 0.1116 |
| Upper Rice Lake | 0.1420 | 0.1141 | 0.0010 | 0.3125 | 0.1088 |
| *Zizania aquatica* | 0.1496 | 0.1313 | 0.0010 | 0.3125 | 0.1107 |

**Table S6.** Estimates of migration rates from BayesAss3-SNPs analysis for a Natural Stand collection and a Cultivated collection of Northern Wild Rice (NWR; *Zizania palustris* L.).

** *See External Excel File ‘Table S6’***

**Table S7.** *D-*statistics (ABBA-BABA) results for a diversity collection of Northern Wild Rice (NWR; *Zizania palustris* L.).

| **P1** | **P2** | **P3** | **ABBA^a^** | **BABA^a^** | **BBAA^a^** | **D** | ***p*-value** | **F4-ratio** | **Z^b^** |
| --- | --- | --- | --- | --- | --- | --- | --- | --- | --- |
| Cultivated | Natural Stands I | Natural Stands II | 348.181 | 336.828 | 367.406 | 0.017 | 0.098 | 0.193 | 1.655 |

^a^ Number of ABBA and BABA sites. *Z. aquatica* was used as an outgroup for comparisons.

^b^ Z: Z-score

**Table S8.** Significant values for Tajima’s D, *F_ST_*, and XP-CLR scores for a diversity collection of Northern Wild Rice (NWR; *Zizania palustris* L.) based on 5,955 single nucleotide polymorphism (SNP) markers generated via genotyping-by-sequencing (GBS).

*See External Excel File ‘Table S8’*
